# Direct Identification of Proteolytic Cleavages on Living Cells Using a Glycan-Tethered Peptide Ligase

**DOI:** 10.1021/acscentsci.2c00899

**Published:** 2022-10-11

**Authors:** Kaitlin Schaefer, Irene Lui, James R. Byrnes, Emily Kang, Jie Zhou, Amy M. Weeks, James A. Wells

**Affiliations:** † Department of Pharmaceutical Chemistry, University of California San Francisco, San Francisco, California 94158, United States; ‡ Department of Cellular and Molecular Pharmacology, University of California San Francisco, San Francisco, California 94158, United States

## Abstract

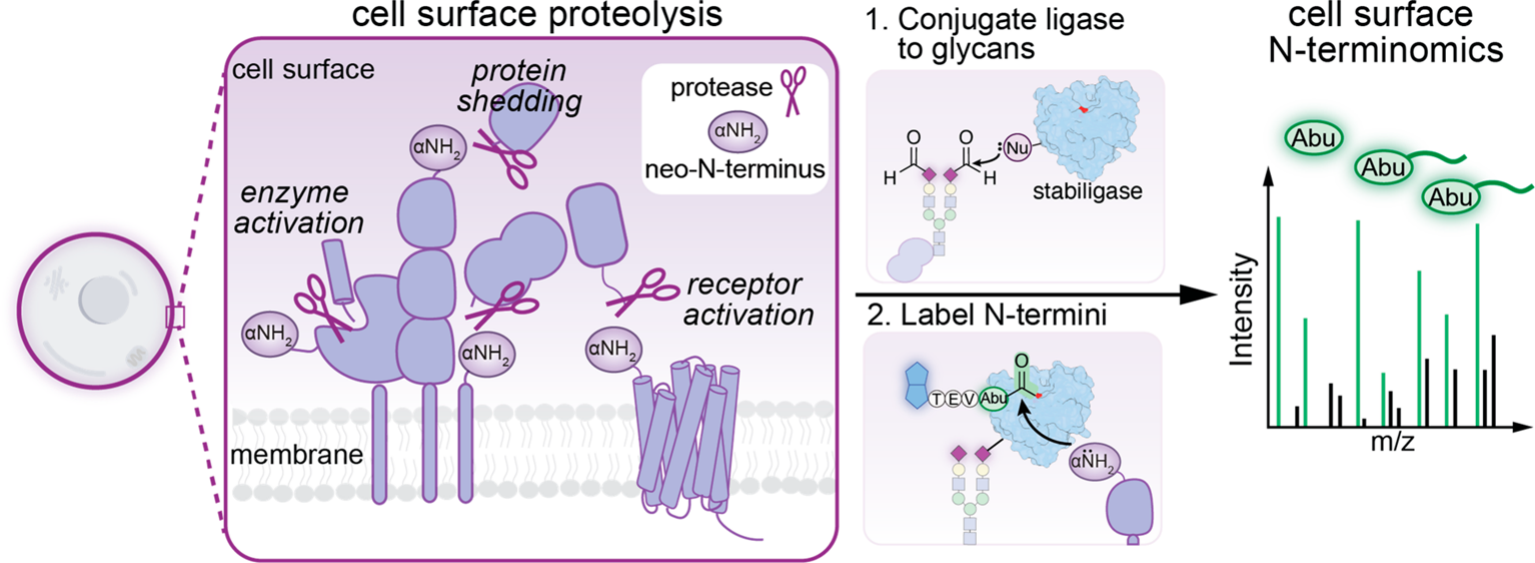

Proteolytic cleavage of cell surface proteins triggers critical processes including cell–cell interactions, receptor activation, and shedding of signaling proteins. Consequently, dysregulated extracellular proteases contribute to malignant cell phenotypes including most cancers. To understand these effects, methods are needed that identify proteolyzed membrane proteins within diverse cellular contexts. Herein we report a proteomic approach, called cell surface N-terminomics, to broadly identify precise cleavage sites (neo-N-termini) on the surface of living cells. First, we functionalized the engineered peptide ligase, called stabiligase, with an N-terminal nucleophile that enables covalent attachment to naturally occurring glycans. Upon the addition of a biotinylated peptide ester, glycan-tethered stabiligase efficiently tags extracellular neo-N-termini for proteomic analysis. To demonstrate the versatility of this approach, we identified and characterized 1532 extracellular neo-N-termini across a panel of different cell types including primary immune cells. The vast majority of cleavages were not identified by previous proteomic studies. Lastly, we demonstrated that single oncogenes, *KRAS(G12V)* and *HER2*, induce extracellular proteolytic remodeling of proteins involved in cancerous cell growth, invasion, and migration. Cell surface N-terminomics is a generalizable platform that can reveal proteolyzed, neoepitopes to target using immunotherapies.

## Abstract 2

A proteomic method captures extracellular proteolysis by chemically tethering an engineered peptide ligase to naturally occurring glycans and then enzymatically labeling neo-N-termini on living cells.

## Introduction

The cell surface proteome comprises approximately 3000 proteins that allow a cell to sense its environment, receive extracellular signals, interact with neighbors, and control cell entry.^6^ Whereas the functional diversity of intracellular proteins is controlled by hundreds of different post-translational modifications (PTMs),^7^ protein modifications in the extracellular space are far more limited. Most common PTMs on cell surface proteins, such as glycosylation and lipidation, are introduced by intracellular enzymes during protein maturation and trafficking. In contrast, proteolysis is a frequent and essential cell surface PTM that is catalyzed outside of the cell by a large repertoire of membrane-bound and secreted proteases. Among their many roles, proteases activate enzymes by removing inhibitory domains, release cytokines, initiate or repress signal transduction, and modulate cell adhesion.^8^ As a result, aberrant proteolysis contributes to many pathological states including inflammatory diseases and most cancers.^9^ Interrogating the biological roles of extracellular proteases requires knowledge of cleavage events, and many of these remain either ill-defined or uncharacterized.

Advances in mass spectrometry (MS) have greatly improved the global identification of proteolysis, but extracellular proteolytic modifications remain challenging subjects to characterize with current techniques.^10−13^ A common approach is to isolate proteins that are proteolytically shed, otherwise called the secretome, into the supernatant of cell cultures.^14^ Although this method generates substantial information regarding shed proteins, it does not precisely identify cleavage sites and is primarily limited to proteins cleaved close to or within the membrane. Another approach is to enrich and identify C- and N-proteolytic termini peptides from whole cell lysates.^12,15−17^ The high complexity of the proteome and the challenging properties of many membrane proteins—most frequently poor solubility and low abundance relative to intracellular proteins—lead to incomplete coverage of extracellular proteolysis using these approaches. Recently we demonstrated that genetically encoding a membrane-anchored subtiligase enhanced the labeling and identification of cell surface neo-N-termini, but the utility of this method was limited by the need for cellular engineering.

Here we describe a general chemical ligation strategy that tethers subtiligase to glycans on the surface of living cells and enables efficient labeling of cell surface neo-N-termini without genetic manipulation. By installing glycan-tethered ligase on a range of cell types, including both immortalized adherent cells and primary immune cells, we profiled a total of 1532 neo-N-termini across 449 diverse membrane proteins, the vast majority of which were not previously annotated. Lastly, we coupled cell surface N-terminomics with quantitative proteomics and uncovered how prominent oncogenes, *KRAS(G12V)* and *HER2*, induce extracellular remodeling through proteolysis. Compatible across cellular contexts, cell surface N-terminomics has the potential to greatly accelerate our discovery of proteolytic neoepitopes for immunotherapeutic approaches.

## Results and Discussion

### Attaching Stabiligase to Native Glycans Enables Efficient Labeling of Cell Surface Neo-N-Termini

**Figure 1:**
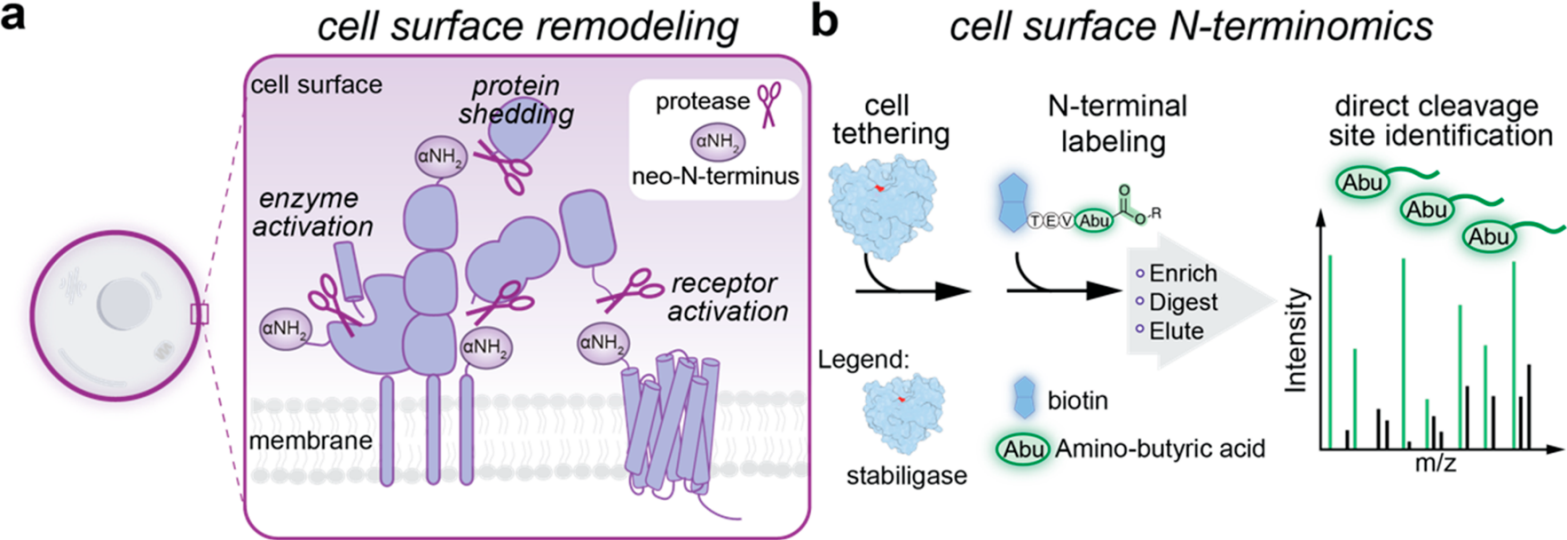
Chemoenzymatic approach (cell surface N-terminomics) for characterizing cell surface proteolytic modifications on living cells. (a) Extracellular proteases (scissors) regulate fundamental cellular processes by cleaving the extracellular domains of proteins. These cleavage events often create new N-termini (neo-N-termini) on the cell surface. (b) To broadly capture proteolysis within the native membrane environment, we envisioned a mass-spectrometry-based approach in which an engineered peptide ligase (stabiligase) is chemically tethered to cell surfaces. In the presence of accessible N-termini, stabiligase labels α-amines with a peptide ester containing a biotin (blue), a TEV-protease cleavage site, and an amino-butyric acid mass tag (Abu).^5^ Following an MS workflow (neutravidin enrichment, proteolytic digestion, and release from neutravidin), Abu-tagged N-termini peptides (green) are identified using LC-MS-MS analysis.

N-Terminomics is a powerful proteomics technology based on subtiligase, a mechanistically engineered ligase that can specifically label N-terminal α-amines of proteins in a complex milieu (Figure 1b).^18,19^ The basic activity of subtiligase is to catalyze peptide ligation between a donor peptide with a C-terminal ester and the N-terminal amine of an acceptor oligopeptide or protein. The ligase and its further engineered variants are used for diverse biotechnological applications, including peptide cyclization and protein synthesis.^20^ In N-terminomics, the ligase typically labels target proteins with a short peptide comprising a biotin handle, a TEV-protease cleavage site, and an aminobutyric-acid (Abu) mass tag (Figure S1).^20,21^ In brief, biotinylated proteins are enriched and proteolytically digested, and the N-terminal peptides are identified after LC-MS-MS analysis by the Abu-mass tag (Figure 1b). While subtiligase has efficiently captured intracellular proteolytic substrates,^22^ we rarely identified cell surface neo-N-termini when labeling either intact cells or cell lysates.^23^ As a solution, we genetically encoded a transmembrane (TM)-subtiligase in HEK293T cells that displayed on the cell surface and efficiently labeled membrane proteins. Although this method identified hundreds of extracellular neo-N-termini, it required cellular engineering that restricted further applications.

**Figure 2:**
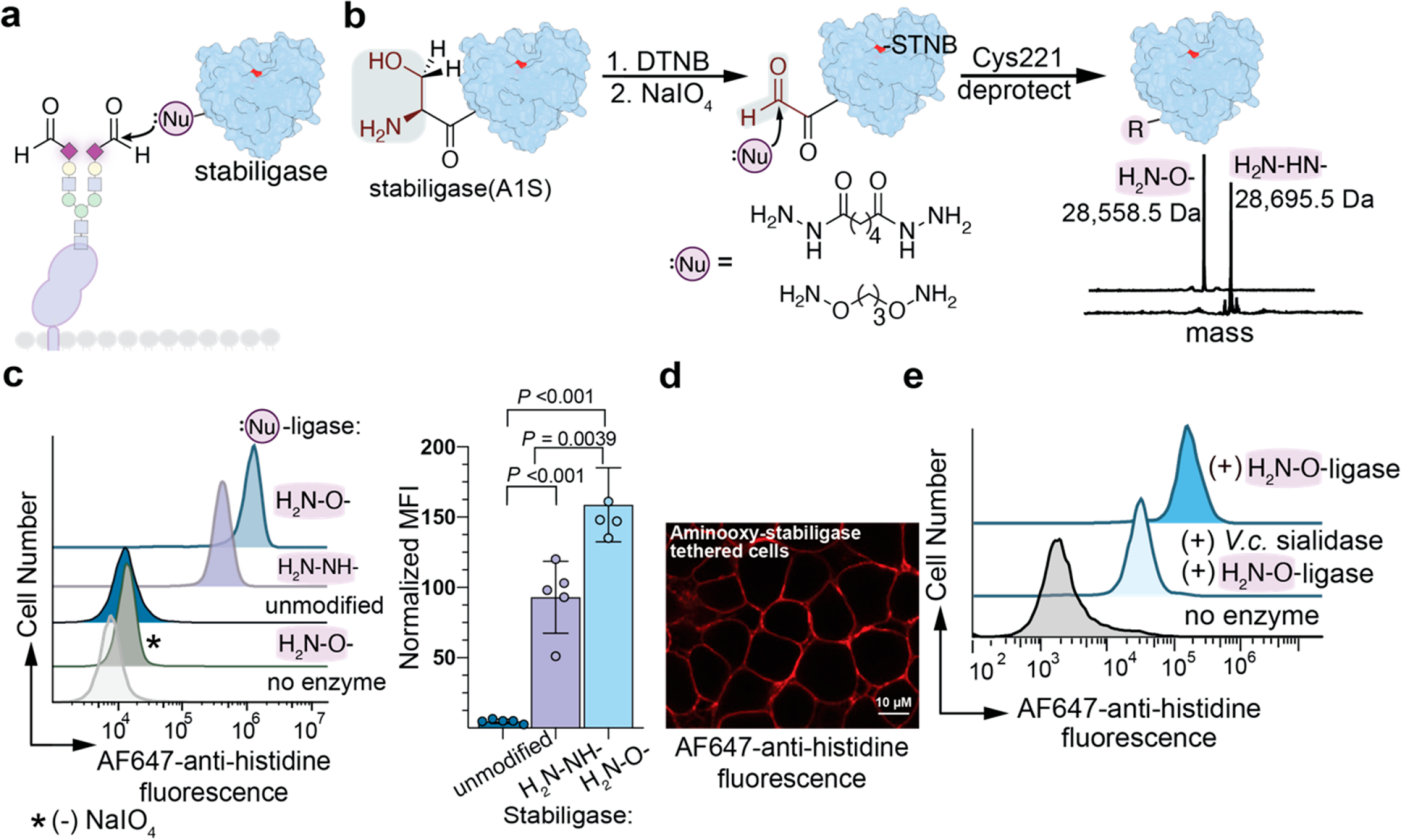
An N-terminal nucleophile on stabiligase mediates covalent attachment to cell surface glycans. (a) Imine-ligation strategy for tethering stabiligase to cells. A brief sodium periodate (NaIO_4_) treatment generates aldehydes on extracellular glycans that may react with nucleophilic species.^1,2^ (b) Synthetic scheme for conjugating a nucleophile onto the N-terminus of stabiligase(A1S). Sodium periodate treatment generates an N-terminal aldehyde on a thiobenzoate-modified (TNB) stabiligase(A1S) (5 min, 4 °C), which is then coupled to a bis-aminooxy- or bis-hydrazide reagent (overnight, 4 °C). Lastly, the sulfhydryl active site (Cys221) is freed upon the addition of a reducing agent (TCEP). Fully functionalized stabiligase(A1S) was obtained as evident by ESI-MS TOF analysis. (c) HEK293T cells treated with NaIO_4_ (10 min, 4 °C) were then incubated with the nucleophilic stabiligases and the catalyst aniline (15 min, 5 μM stabiligase, 4 °C). As determined by flow cytometry, the N-terminal nucleophile enabled stable and dramatically improved levels of stabiligase attachment to cells relative to the unmodified enzyme. (d) Fluorescence microscopy showed exclusive cell surface display using aminooxy-stabiligase. (e) HEK293T cells were treated with *Vibrio cholerae* (V.c.) sialidase prior to tethering aminooxy-stabiligase. Trimming glycans reduced stabiligase attachment as determined by flow cytometry. Data are presented as the mean ± s.e.m., and *P* values were calculated using one-way ANOVA followed by Tukey’s multiple comparisons test.

We envisaged a generalizable cell surface N-terminomics approach to characterize proteolysis sites across cell types without requiring genetic manipulation (Figure 1). To achieve this, we hypothesized that glycans, omnipresent on cell surfaces, would allow us to chemically attach subtiligase (Figure 2a). We considered using an imine-ligation strategy; glycan sugars containing diols, particularly sialic acid, are sensitive to mild periodate oxidation and form aldehydes that can be coupled to aminooxy- and hydrazide-nucleophiles (Figure 2a).^1,2^ First, we designed a conjugation strategy to append an α-nucleophile on the N-terminus of stabiligase (Figure 2b). Auto-prodomain removal during protein expression generates an N-terminal alanine (A1) on mature stabiligase; to site-selectively modify the ligase, we mutated A1 to serine (A1S) and created a vicinal α-amino-alcohol. This mutation did not alter expression or purification of stabiligase (Figure S2). A brief sodium periodate incubation (NaIO_4_; 5 min, 4 °C) was sufficient to convert the N-terminal amino-alcohol to a glyoxyl aldehyde (Figure S2). However, we also observed a minor product consistent with the oxidation of the active site cysteine, C221. To prevent reduced activity, we treated stabiligase(A1S) first with Ellman’s reagent (5,5′-dithiobis(2-nitrobenzoic acid; DTNB) and generated a disulfide-protected, TNB-C221 adduct.^24^ We then oxidized the TNB-protected ligase, introduced the α-nucleophile during an overnight incubation with either a bis-aminooxy- or bis-hydrazide-reagent, and lastly removed the TNB group from C221 with a reducing agent (Figure 2b, Figure S2). This strategy resulted in stabiligase fully conjugated with either an N-terminal aminooxy or hydrazide group.

To pilot stabiligase attachment to cells, we treated HEK293T cells with sodium periodate (10 min, 4 °C) to form aldehydes on glycans^1,2^ and then incubated them with either of the two conjugated stabiligases and an amine catalyst (aniline; 15 min, 4 °C).^14,15^ Robust tethering of both α-nucleophilic stabiligases was determined by flow cytometry, although significantly higher levels of attachment were observed for aminooxy-stabiligase under these conditions (Figure 2c), consistent with higher kinetic rates of aminooxy-nucleophiles.^25^ Importantly, both the α-nucleophile conjugate and periodate treatment on cells were necessary for stabiligase attachment (Figure 2c). Furthermore, we visualized HEK293T cells stained with an AlexaFluor647-antihistidine antibody, which monitors the C-terminal histidine tag on stabiligase, and fluorescence microscopy confirmed that the aminooxy-stabiligase was indeed anchored to the membrane (Figure 2d). To assess tethering specificity, we pretreated cells with *Vibrio cholerae* sialidase,^2,26^ a hydrolase that trims the terminal glycan sugars, prior to tethering, and observed reduced levels of aminooxy-stabiligase on cells (Figure 2e). We conclude that stabiligase modified with an N-terminal α-nucleophile stably attaches to cells through oxidized glycans.

Alternate methods for covalent attachment of stabiligase to the cell surface were also considered. Using an aminooxy-propargyl reagent, we functionalized the N-terminus of stabiligase(A1S) with an alkyne to explore a click-chemistry route. We grew HEK293T cells in media supplemented with peracetylated GalNAz to metabolically incorporate azido-groups into extracellular glycans and then attempted tethering with alkynyl-stabiligase under click conditions suitable for living cells.^27,28^ In comparison to N-terminal nucleophilic stabiligases, modest attachment of alkynyl-stabiligase was determined by flow cytometry (Figure S3). Given this result, we moved forward with an imine-ligation strategy to display stabiligase on the cell surface.

**Figure 3:**
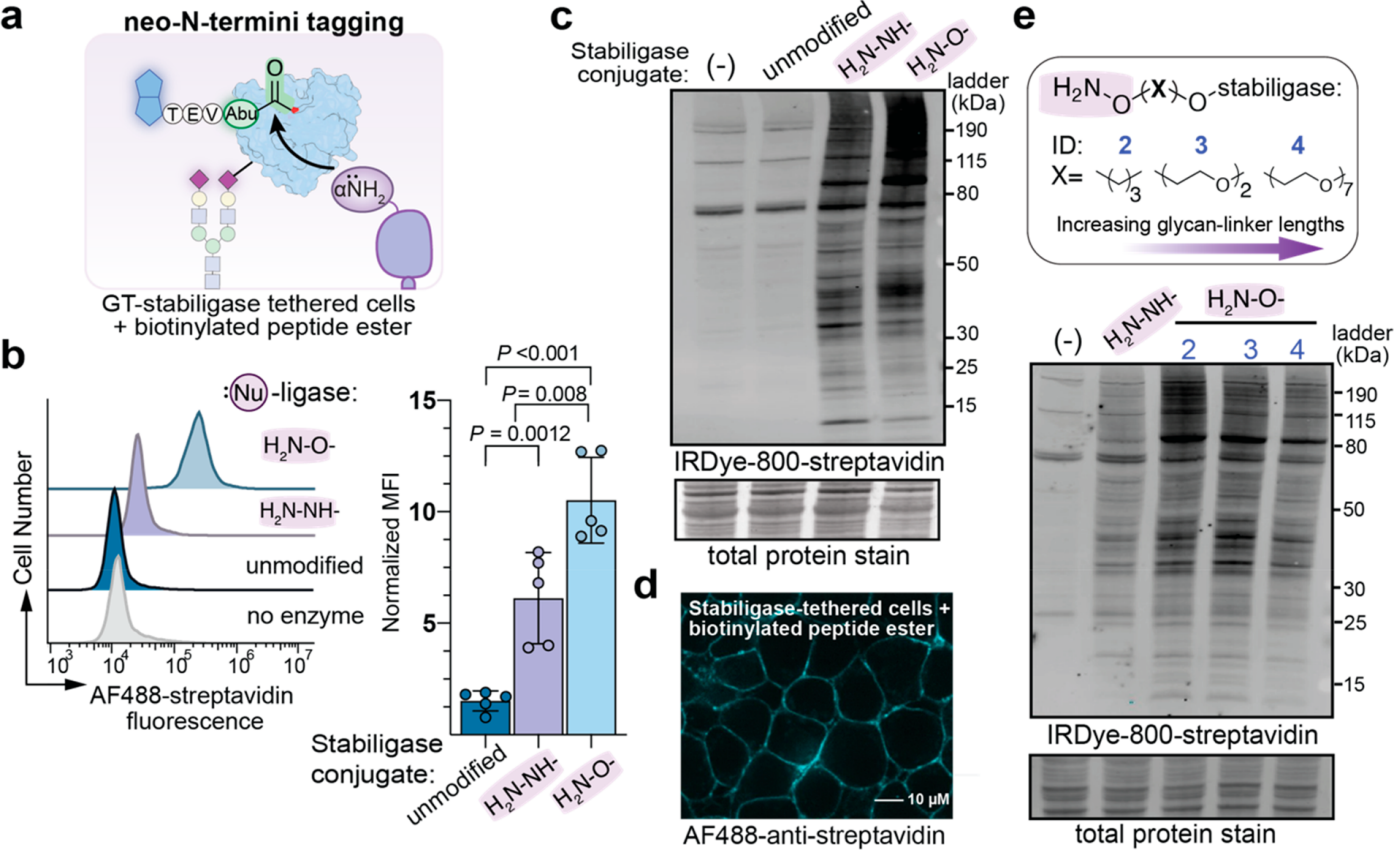
Glycan-tethered stabiligase efficiently labels cell surface proteins. (a) In the presence of a biotinylated peptide ester, stabiligase forms a thioester adduct that reacts with N-terminal amines of proteins. (b) Cells were briefly treated with the biotinylated peptide ester (15 min, 25 °C) for flow cytometry analysis. Cells with GT-stabiligase showed dramatically improved biotinylation compared to those incubated with unmodified stabiligase and the peptide ester. (c) Proteins in the membrane fractions of cells were analyzed by Western blot, and biotinylation intensities were consistent with flow cytometry analysis. Comparing the membrane and the cytosolic fractions by Western blot analysis showed biotinylation predominantly in membrane proteins (Figure S4). (d) Fluorescence microscopy of cells tethered to GT-stabiligase and then treated with the biotinylated peptide ester showed cell surface biotinylation. (e) GT-stabiligases with longer linkers, which increase the theoretical distance between the glycan anchor and the ligase domain, showed slightly reduced activity as evaluated by Western blot. Data are representative of at least three independent experiments with similar results. In panel b, data are presented as the mean ± s.e.m., and *P* values were calculated using one-way ANOVA followed by Tukey’s multiple comparisons test.

We then assessed ligase activity of glycan-tethered (GT)-stabiligase displayed on HEK293T by incubating cells with a biotinylated peptide ester substrate for 15 min at room temperature (Figure 3a). Flow cytometry analysis showed that biotinylation was significantly higher for cells tethered with α-nucleophilic stabiligases compared to cells incubated with an unmodified stabiligase and the peptide ester (Figure 3b). Cytoplasmic and membrane cellular fractions were isolated for Western blot analysis with streptavidin. Biotinylated proteins were observed almost exclusively in membrane fractions from GT-stabiligase labeling, and intensities were congruent with flow cytometry results (Figure 3c, Figure S4). Likewise, fluorescence microscopy of HEK293T cells stained with AlexaFluor488-streptavidin further showed that N-terminal labeling took place along the cell membrane (Figure 3d). Cell toxicity was evaluated after peptide ligation, and we observed only a modest decrease in cell viability (15%; Figure S5). Collectively, these data show that the GT-stabiligase broadly labels cell surface proteins, and that aminooxy-functionalized stabiligase is a better conjugate for cell surface N-terminal ligation. We were also curious as to whether the proximity of the stabiligase domain to the glycan affected ligation and prepared two additional GT-stabiligases with an N-terminal aminooxy group attached via a 2 or 7 poly(ethylene glycol) (PEG) linker unit. Although these alternative conjugates add flexibility and theoretical distance between the glycan anchor and ligase domain, we observed slightly decreased N-terminal labeling with longer-linked stabiligases and moved forward using the propanyl-linked aminooxy-stabiligase (Figure 3e).

### Cell Surface N-Terminomics Captures Neo-N-Termini Across Different Cell Types

**Figure 4:**
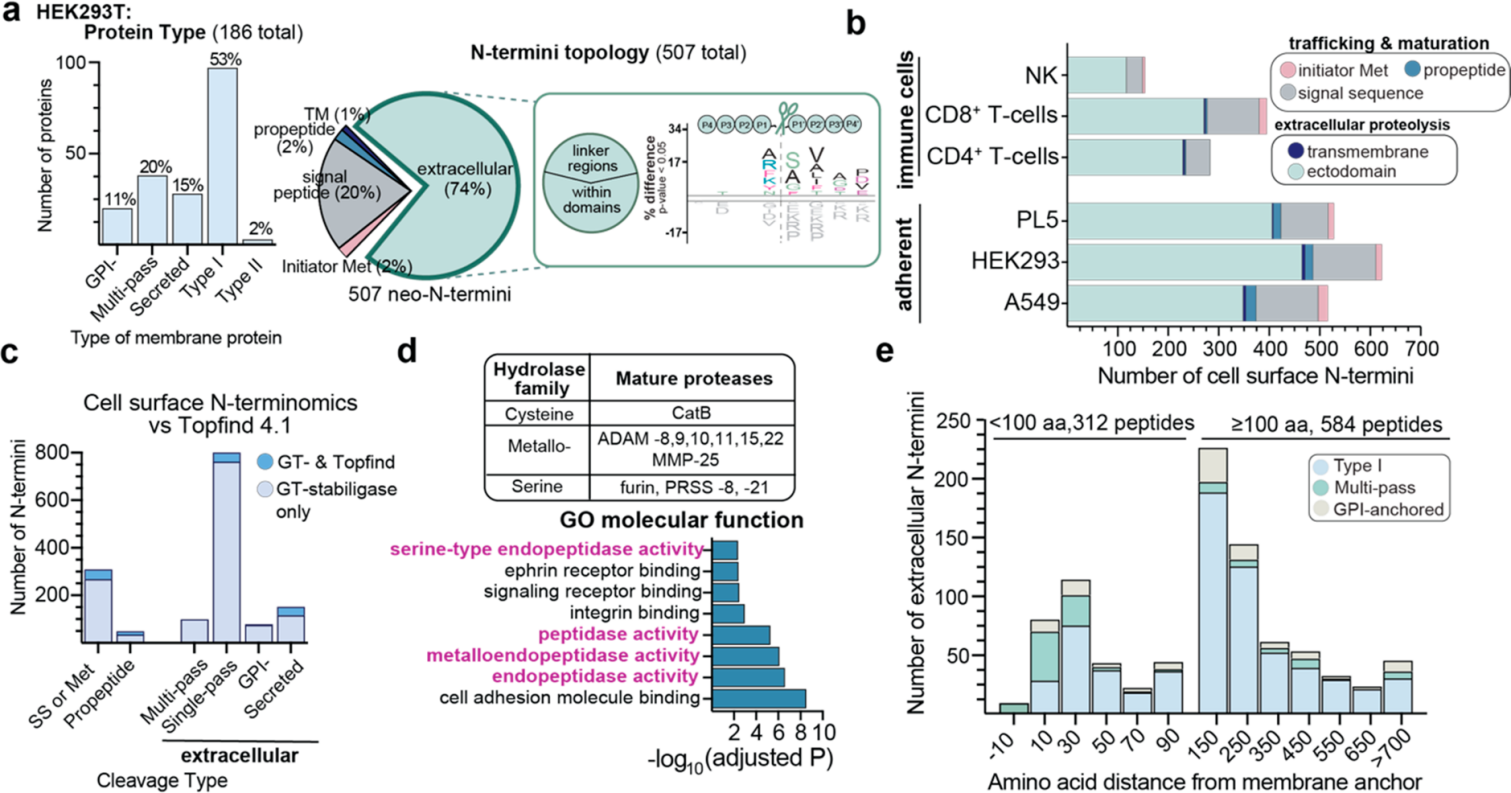
Cell surface N-terminomics broadly captures neo-N-termini across different cell types. (a) Initial cell surface N-terminomics on HEK293T cells yielded 507 cell surface N-termini mapped to 186 proteins. Different types of membrane proteins represented by neo-N-termini were distributed similarly to the population ratios. Neo-N-termini were also grouped based on the cleavage topology: initiator methionine (Met), signal peptide, propeptide junction, within a transmembrane helix (TM), and extracellular domains of proteins. The vast majority of peptides (74%) mapped to extracellular domains of proteins and were localized either in linker regions or within domains that were predominantly beta-strands (see also Figure S7). An iceLogo visualization of the P4–P4′ residues flanking the cut-site (scissors) shows a range of amino acids at the P1 position. (b) Cell surface N-terminomics was then extended to additional cell types, including immortalized adherent cell types and primary immune cells, and we identified hundreds of cell surface neo-N-termini for each cell type. (c) These peptides were compared to N-termini represented in the Topfind 4.1 knowledge base, and only a small percentage of neo-N-termini were reported previously (dark blue).^4^ (d) Gene ontology (GO) analysis of proteins identified with promature junction cleavages showed an over-representation of proteases. These include endopeptidases belonging to diverse hydrolase families with different substrate profiles. (e) To approximate the distance between the proteolytic site and the cell membrane, extracellular cleavages were distributed based on the number of amino acids between the membrane anchor (either the proximal TM helix or GPI-anchor) and the neo-N-termini. See also Tables S1 and S2 for proteomic data sets.

Robust GT-stabiligase labeling of membrane proteins on HEK293T cells encouraged us to pursue N-terminomics experiments. In pilot experiments, we tethered GT-stabiligase to preoxidized HEK293T and then labeled cells with the biotinylated peptide ester as described above. Labeled proteins were enriched using neutravidin, digested on-bead with trypsin, and lastly incubated with TEV-protease to release the mass-tagged (Abu) N-terminal peptides for LC-MS-MS analysis. Using features retrieved from the UniProt knowledge database,^29^ we identified 507 Abu-tagged peptides (neo-N-termini) that mapped to extracellular topology within membrane proteins, extracellular secreted proteins, or GPI-anchored proteins localized in the plasma membrane (Figure 4a). Among the proteins observed via N-terminal peptides, most proteins were type 1 single-pass proteins (53%), which is not surprising since type 1 membrane proteins comprise the majority of cell surface proteins and display an extracellular N-terminus available to both native extracellular proteases and GT-stabiligase.^6^ We also identified neo-N-termini corresponding to multipass proteins (20%), secreted proteins (15%), and GPI-anchored (11%) proteins. In contrast, only a few cleavages were observed in type II membrane proteins (2%) which are oriented with a cytoplasmic N-terminus. We repeated the experiment using aminooxy-(PEG)_7_-stabiligase and observed similar numbers of cell surface peptides (407 neo-N-termini) which further supports the notion that GT-stabiligase is flexibly incorporated into the cell surface proteome. These data indicate that there is sufficient length, flexibility, and mobility in the membrane for GT-stabiligase to access neo-N-termini, and hereafter, we use only the original aminooxy-stabiligase for cell surface N-terminomics.

Further analysis showed that identified neo-N-termini were distributed across several types of proteolytic events: the removal of initiator methionine, signal peptide cleavage, propeptide removal, and postmaturation cleavage within the extracellular regions (Figure 4a). The majority of N-termini (74%) mapped to the latter group and represent potential cleavage sites of extracellular proteases. Alignment of residues (P4–P4′) flanking these inferred cleavage sites did not reveal a significant consensus sequence around the scissile bond (Figure 4a), which suggests, not surprisingly, that multiple proteases are responsible for generating these neo-N-termini. We also considered the protein structure at extracellular cleavage sites; the neo-N-termini mapped predominantly to either interdomain, disordered regions, or beta-strand regions within domains, consistent with proteolytic substrate preferences (Figure S7).^30^

We then performed cell surface N-terminomics on a panel of different cell types that included adherent cells and primary immune cells (Figure 4b). Across the six cell types tested, we observed hundreds of N-termini, ranging from 500 to 600 for adherent cell lines and 200–400 for immune cells. As seen with HEK293T cells, the majority of extracellular neo-N-termini observed were generated by postmaturation cleavages (mean, 74%) while the remainder were predominantly signal sequence cleavages. In total, there were 1532 cell surface neo-N-termini from 449 cell surface proteins (Figure 4b). Notably, multiple closely spaced cleavage sites were observed within some proteins. To better characterize how many functionally unique cleavages were observed within proteins, we grouped closely spaced cleavages (less than three residues apart) and observed 936 unique cleaved regions on 449 cell surface proteins. An over-representation analysis based on gene ontology (GO) annotations was explored for proteins with proteolytic extracellular neo-N-termini, and specific cellular processes were enriched for adherent cells and primary immune cells (Figure S7).^31^ Although this finding is anticipated based on the underlying biological differences between the cell types tested, it highlights the value of profiling extracellular proteolysis across cellular contexts.

We also assessed how cell surface N-terminomics compared to other proteomics methods. Topfind 4.1 is a database containing experimentally observed N-termini using various proteomic approaches (e.g., subtiligase lysate labeling,^18^ N-TAILs,^12^ and COFRADIC^11,15^). To compare our results to Topfind 4.1, we grouped N-termini identified by GT-stabiligase based on cleavage type and subdivided extracellular proteolysis sites according to the type of membrane protein they mapped to.^4^ Strikingly, only 143 N-termini in our data (∼9%) were also found in the Topfind 4.1 database. Even among the well-annotated protein maturation cleavages identified by GT-stabiligase (i.e., those identified in Uniprot as signal peptide removal, propeptide cleavage, etc.), only a small percentage were previously characterized by other methods. We also noted that ∼50% of the shared N-terminal peptides originated from extracellular regions of single-pass or secreted proteins, whereas no cleavage sites within multipass proteins were found in Topfind 4.1. We then compared our data to the CSPA (Cell Surface Protein Atlas) project, which used cell surface capture (CSC) proteomics to identify 1492 cell surface proteins across 41 human cell types.^1,32^ As expected, we observed significant overlap in proteins between GT-stabiligase N-terminomics and CSPA (67%). Based on Uniprot annotations of N- and O-glycosylation sites, we found that proteins uniquely identified by GT-stabiligase were predicted to be modestly glycosylated (median, 2 glycosites) compared to shared proteins (median, 5 glycosites). We speculate that these proteins were not identified in CSPA because CSC proteomics requires glycosylation for enrichment whereas surface-anchored GT-stabiligase may label neighboring proteins. These comparisons further demonstrate the notion that GT-stabiligase yields broad coverage of N-termini on the cell surface with distinct utility relative to other methods.

N-Terminomics with GT-stabiligase also gives several lines of evidence as to which proteases are present and active on the cell surface. Proteases are commonly synthesized as inactive precursors that require the removal of an inhibitory N-terminal propeptide for activation.^33^ For proteins identified with promature junction cleavages (57 neo-N-termini), we observed an over-representation of proteins with endopeptidase activities based on GO analysis (Figure 4d).^31^ In total, we observed 11 mature, extracellular proteases from several hydrolase families, including seven metalloproteases. The latter group contains 4 catalytically active ADAMs, dedicated sheddases that cleave proteins within their juxtamembrane region,^14^ and we thought that their activity should be reflected in the N-terminomics data. To estimate how many shed proteins were observed, we approximated the physical distance of the cleavage sites to the membrane through amino acid distances. About 140 cleavage sites were located within 30 amino acids of the membrane and are considered candidate shed proteins (Figure 4e). Consistent with this hypothesis, we observed well-studied examples of shed proteins including Notch (e.g., Notch 1,2),^34^ receptor kinases (e.g., PTK7, PTPRK),^35^ syndecans (e.g., SDC-1, SDC-4),^36^ and cell surface receptors (e.g., CD99, CD44, CCR6).^14^

Membrane-proximal shedding is a subset of extracellular proteolysis, and more than half of observed extracellular neo-N-termini were located further than 100 amino acids from the membrane (Figure 4e). Concurrently, we also observed activated proteases that are not typically considered sheddases. To better characterize extracellular neo-N-termini, we determined structural features surrounding the cleavage sites: relative domain distances, predicted secondary structure, and solvent accessibility (Figure S7). Similar to initial studies with HEK293T cells, neo-N-termini localized to solvent-exposed regions and primarily unstructured or beta-strand regions. We also observed inter- and intradomain cuts across all extracellular neo-N-termini. Examples of previously characterized cleavages between domains include cleavages between the ephrin-binding domain and fibronectin domains of Eph A2 and B2, and proteolysis between the light and heavy chains of the urokinase plasminogen activator.^37,38^ Precise intradomain cleavages were also identified, including the established furin-cleavage within the Sema domain of the RON kinase receptor and autoproteolytic site within the GPS domains for two adhesion GPCRs (AGR2 and ADGR6).^29,39,40^ Across all single-pass membrane proteins, over half of neo-N-termini (65%) were located between the first and last extracellular domain (Figure S7). Although these events are not membrane-proximal shedding events, the position of these N-termini suggests that they may have significant structural impact.

### Single Oncogenes Induce Proteolytic Remodeling of the Cell Surface

Cellular disease states are commonly associated with dysregulated proteolytic modifications, but identifying and quantifying the cleavages induced by specific oncogenes remains challenging. We previously quantified oncogene-induced changes in the surface expression of membrane proteins using an immortalized, nontumorigenic cell line (MCF10A) transformed with individual oncogenes.^3,41^ Two oncogenes, *KRAS(G12V)* and *HER2*, contributed to significant alterations to the cell surface proteome through changes in both protein expression and glycosylation, and we wondered if these transformations might also alter the proteolytic landscape. Importantly, we previously found that CSC proteomics was not biased by glycan alterations.^3^ Using flow cytometry, we first assessed whether glycan variations may affect the tethering of GT-stabiligase or peptide ligation. Encouragingly, no significant differences were observed among the parent MCF10A transduced with an empty vector (ev) and the two oncogenic cell lines (Figure S8).

**Figure 5:**
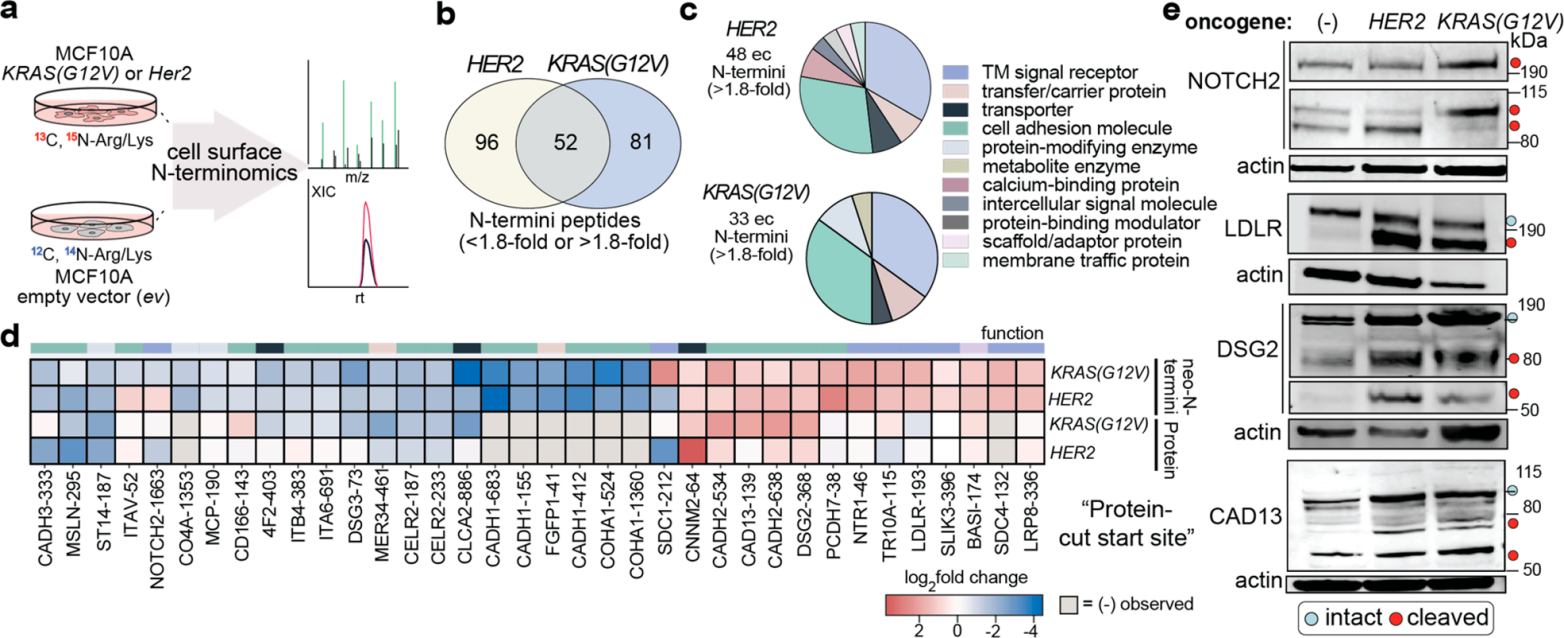
Single oncogenes, HER2 and KRASG12 V, drive common and unique proteolytic cleavages on cell surfaces. (a) Cell surface N-terminomics was performed on SILAC-labeled MCF10A cell lines carrying common oncogenes, HER2 or KRAS(G12 V), and an empty vector (ev) parental cell line. (b) Differentially abundant neo-N-termini (1.8-fold threshold) were observed in both oncogenic data sets and also individual oncogenic backgrounds (see also Figure S9). (c) Upregulated cleavage sites within extracellular (ec) regions were mapped predominantly to TM-signal receptors and cell adhesion proteins, as represented by protein family annotations. (d) A heat map shows comparisons between shared extracellular neo-N-termini observed in the presence of HER2 or KRAS(G12 V) and corresponding CSC-based protein enrichments reported previously.^3^ (e) Representative Western blot detection of full-length (blue) and cleaved proteoforms (red) of cell surface proteins [NOTCH2, DSG2, LDLR, and Cadherin-13 (CAD13)] that were consistent with quantitative proteolytic differences observed using cell surface N-terminomics. Of note, NOTCH2 is cleaved by furin (S1 site) which generates two protein fragments: a large extracellular NOTCH2 fragment (top band) which associates with another NOTCH2 consisting of a small extracellular portion and a large intracellular region (middle band). The ADAM-protease cleavage at the S2 site results in the third NOTCH2 fragment (lowest band) which likely overlaps with the cleavage product of γ-secretase (S3 site). We would not expect to see the S3 cut by cell surface N-terminomics because the neo-N-terminus is intracellular. Individual experiments were performed in triplicate with similar results. See also Table S3 for proteomic data sets.

For quantitative N-terminomics, MCF10A cell lines were cultured in stable isotopic labeling of amino acid (SILAC) media. The oncogene-transformed [*HER2* or *KRAS(G12V)*] cell lines were combined with parental MCF10A cells transformed with an ev, labeled with GT-stabiligase, and incubated with the peptide ester as described above (Figure 5a). N-Terminomics was performed on five biological replicates for both oncogene sets. From these we quantified 303 N-terminal peptides mapped to 151 proteins and observed 233 neo-N-termini on 89 proteins with differential abundances (1.8-fold threshold). Among these N-termini, 35–40% of extracellular neo-N-termini overlapped between the *HER2*-overexpression and *KRAS(G12V)* data sets, and shared peptides were similarly enriched or depleted (Figure 5b). In both oncogenic-transformations, as shown in Figure 5c, enriched extracellular neo-N-termini predominantly mapped to cell adhesion proteins and transmembrane signal receptors, two pathways reportedly modulated by proteases.^35,42^

Next, we assessed whether changes in cell surface N-termini coincided with differences in protein abundance in the presence of either oncogene. We plotted the fold-changes of extracellular neo-N-termini alongside fold-changes in surface expression, as previously determined by CSC proteomics (Figure 5d, Figure S9). As shown in Figure 5d, 52 neo-N-termini with a greater than 1.8-fold-change in abundance mapped to 31 proteins. 80% of these proteins were observed by CSC, and interestingly, the protein abundance ratios were modestly correlated with N-termini abundance. Similar observations were made for comparisons with individual oncogene data sets (Figure S9). We note that proteolytic removal of large extracellular domains may contribute to contradictory changes. For instance, syndecan-4 (SDC4) shedding is highly upregulated in both oncogene data sets, and the protein was not observed in CSC proteomics. It is likely that cleavage leaves behind a juxtamembrane neo-N-terminus not suitable for CSC identification. Transcript levels for SDC4, however, were not significantly altered in the presence of *KRAS(G12V)* suggesting that regulation is at the level of the protease.^41^ Proteases may be differently expressed under oncogenic transformations, but other factors such as protein interactions and additional PTMs may also influence cleavage events.

To provide additional validation, we performed Western blot analysis of selected proteins detected by both CSC and N-terminomics in the parent and transformed cell lines. These experiments used commercially available antibodies that recognize both the full-length and cleaved proteoforms (Figure 5e). Notch2 is a receptor and transcription factor in adjacent-cell signaling pathways that is activated by a series of proteolytic cleavages. Mature Notch2 is first cleaved by a furin-like convertase in the Golgi (S1 site), and once on the cell surface, ligand-binding induces membrane-proximal cleavage by an ADAM metalloprotease (S2) followed by cleavage within the membrane by γ-secretase (S3).^34^ In the parent and both transformed cell lines, we observed N-terminal peptides from both S1 and S2 sites and could observe their cleavage products by Western blot. In *KRAS(G12V)* cells, cleavage at S1 increased with concurrent diminished cleavage at S2; in contrast, only an enriched S2 cleavage site was observed in *HER2*-expressing cells. For both cleavage sites, the N-termini ratios were in good agreement with the protein intensities visualized by Western blot analysis. We analyzed three other proteins of interest: DSG2, LDLR, and Cadherin-13. Proteolysis of cell-adhesion protein DSG2 plays a role in both cancer and inflammatory cells,^43^ and the neo-N-termini characterized here map to domains reportedly cleaved by metalloproteases and ADAM-proteases.^43^ Western blot analysis showed two intense bands beneath the intact DSG2 protein in lysates of *KRAS(G12V)* and *HER2*-expressing cells consistent with neo-N-termini locations. The LDLR receptor is involved in lipid homeostasis among other functions.^44^ The enriched neo-N-terminus of LDLR was observed in both oncogene data sets and matches a previous report of a metalloprotease cleavage site that results in loss of LDL-class A ligand binding domains 1–4.^44^ In agreement with these data, we observe strong protein signal for a species of a molecular weight consistent with the expected product of the cleavage event. Lastly, we observed enriched N-termini mapped to propeptide and extracellular cleavages of a GPI-linked cadherin called Cadherin-13 (T-cadherin) which affects cell migration in various cancer types. Similar to our N-terminomics results, we indeed observe increased proteolytic bands consistent with its propeptide-activation and further extracellular cleavage for both *HER2*- and *KRAS(G12V)*-transformed cells. While not exhaustive, these examples and the fact that we find other reported cleavages precisely matching literature reports show that cell surface N-terminomics can accurately capture proteolytic modifications that arise after malignant transformations.

## Conclusion

The proper functioning of human cells requires extensive proteolytic modifications to the cell surface. We present a generalizable proteomic approach to identify those cleavages across human cell types. We first developed a straightforward, site-selective conjugation method to append an N-terminal nucleophile on stabiligase. To achieve broad coverage of neo-N-termini while minimally disrupting living cells, we then developed oxime ligation conditions to covalently tether stabiligase to oxidized glycans. Glycan-tethered stabiligase efficiently labels neo-N-termini of both nonglycosylated and glycosylated cell surface proteins. We validated this strategy in varied cell types, including adherent immortalized lines and primary immune cells, and then explored proteolytic alterations induced by common oncogenes. Collectively, across the initial cell panel and isogenic cells, we identified 1637 unique cell surface N-termini across 507 proteins with diverse structures and functions. From these N-termini, we find evidence that proteases impose marked changes to cell surface proteins that include the shedding of entire extracellular portions, removal of discrete protein domains, or the release of inhibitory domains. While GT-stabiligase was readily introduced in these cell types, cells such as those with sialic acid deficiency may require alternative oxidation strategies. We also note that while cells are exposed briefly to mild sodium periodate oxidation (500 μM NaIO_4_, 10 min, 4 °C), this treatment may result in minor biological effects that ought to be considered when interpreting cell surface N-terminomics data.

Among different cell types examined, cell surface N-terminomics revealed proteolytic modifications in distinct molecular pathways that reflect differences in underlying cell function. A prime example is that we observed proteolytic modifications on proteins enriched in immunological pathways among activated T-cell and natural killer cells. Consistently, extracellular proteases, such as ADAMs, are widely expressed and modulate immunity in immune cells.^45^ Notably, our studies also identified activating cleavages within extracellular proteases from mechanistically diverse families. Although the direct linkage of hydrolase–substrate pairs is challenging due to the complexity of proteolytic networks, cell surface N-terminomics in combination with protease knockouts will be a useful tool for connecting these relationships.

We then employed cell surface N-terminomics on isogenic cell lines expressing the common oncogenes, *HER2* and *KRAS(G12V)*, and found that these transformations influence extracellular proteolysis in shared and distinct ways. This was not surprising as the action of multiple proteases within biological pathways suggests that there is not a specific proteolytic profile common to all cancers.^9^ Under the influence of either oncogene, for example, we observed increased proteolysis of proteins with important roles in cell growth, proliferation, and metastasis. These effects included increased juxtamembrane shedding of syndecan-4 and CD44, which are cut by ADAM- and metalloproteases to release their soluble domains. Concordantly, previous studies suggest that upregulated shedding promotes cancerous proliferation and cell migration.^36,46^ In another example, we observed that *KRAS(G12V)* cells displayed higher levels of proteolytically modified EphA2, a receptor tyrosine kinase that inhibits Ras-induced growth upon ligand-binding. Metalloproteases remove the ligand-binding domain of EphA2 to promote tumor growth at precisely the junction we observed.^47,48^ In contrast to the examples above, several intriguing proteins underwent differential cleavage between the *HER2* and *KRAS(G12V)* transformed cells, most notably Notch2. Notch signaling requires ADAM-protease cutting close to the membrane,^34,49,50^ and we observed upregulation of the corresponding neo-N-terminus in *KRAS(G12V)*-transformed cells and downregulation in *HER2*-expressing cells. Consistently, Notch2 signaling has been reported to be up- or downregulated in different cancer cells and may either promote or suppress tumor growth.^49^ Of note, most oncogene-induced proteolytic modifications we identified were either poorly characterized or previously not annotated. Looking forward, future biochemical and cellular experiments are necessary to understand how cleavage events are regulated and the functional consequences of proteolytic modifications. These studies may also provide insight into how proteins contribute to cancerous phenotypes.

Lastly, cell surface N-terminomics can greatly accelerate our ability to identify proteolyzed neoepitopes for immunotherapies. Recent studies have demonstrated that blocking extracellular proteolysis is an effective strategy for inhibiting tumor growth. For instance, MICA/B proteins are highly shed from the surface of tumor cells, and antibodies that block their proteolysis reduce tumor growth.^51^ We and others have shown that CDCP1, a cell surface receptor, is both upregulated and cleaved in different cancer cells.^41,52,53^ By engineering antibodies that selectively target cleaved CDCP1, we developed an antibody–drug conjugate (ADC) that effectively reduced tumor growth with significantly less toxicity compared to those that recognize both full-length and cleaved proteoforms of CDCP1.^53^ In the future, we envision that cell surface N-terminomics can rapidly identify potential disease-relevant, neoepitopes that arise from dysregulated proteolysis.

## Supporting Information Available

General materials and methods, detailed experimental procedures and protocols, and additional data and figures including a synthetic scheme with corresponding ESI-MS traces, additional biochemical experiments, and additional proteomic analyses and data sets (PDF)
Table S1: Proteomics data corresponding to Figure 4 (XLSX)
Table S2: Topfind 4.1 comparison results (XLS)
Table S3: Proteomic data corresponding to Figure 5 (XLSX)
Table S4: Domain topology information corresponding to Figure S7 (XLSX)
Transparent Peer Review report available (PDF)
