# Supplementary material for: Direct Identification of Proteolytic Cleavages on Living Cells Using a Glycan-Tethered Peptide Ligase
Source: ACS Cent Sci. 2022 Oct 11;8(10):1447–56. doi: 10.1021/acscentsci.2c00899 (PMC9615116; doi:10.1021/acscentsci.2c00899)
Supplement: Supplementary file 1 — oc2c00899_si_001.pdf [file oc2c00899_si_001.pdf]

# Supporting Information

## Direct identification of proteolytic cleavages on living cells using a glycan tethered peptide ligase

Kaitlin Schaefer[1], Irene Lui[1], James R. Byrnes[1], Emily Kang[1,2], Jie Zhou[1], Amy M. Weeks[1,3], James A. Wells[1,4*]

*[1] Department of Pharmaceutical Chemistry, University of California San Francisco, San Francisco, California, 94158, USA.*
*[2]Current Address: Nkarta Therapeutics, 6000 Shoreline Court, Suite 102 South San Francisco, CA 94080*
*[3]Current Address: Department of Biochemistry, University of Wisconsin, Madison, WI*
*[4]Department of Cellular and Molecular Pharmacology, University of California San Francisco, San Francisco, California, 94158, USA.*

*[*]Corresponding Author: jim.wells@ucsf.edu*

**Table of Contents**

p. 2 **Experimental materials and methods**
p. 10 **Figure S1.** Chemical structure of biotinylated peptide ester
p. 11 **Figure S2.** Synthetic schematic and corresponding ESI-MS traces for the N-terminal conjugation of stabiligase
p. 12 **Figure S3.** Tethering approach using alkynyl-stabiligase and metabolic-labeling of glycans
p. 13 **Figure S4.** GT-stabiligase predominantly labels membrane proteins under short labeling incubations.
p. 14 **Figure S5.** Cell viability is not significantly affected by stabiligase tethering or labeling activity.
p. 15 **Figure S6.** Icelogos for neo-N-termini identified at signal peptide or pro-peptide junctions shows signature sequence preferences.
p. 16 **Figure S7.** Characterization of identified extracellular neo-N-termini by GO analysis, surface accessibility, and protein domain architecture.
p. 17 **Figure S8.** Stabiligase tethering and labeling is unaffected by cell surface modifications among different transformed, oncogenic cell lines.
p. 18 **Figure S9.** Heat map comparing cell surface N-termini and surfaceomics enrichment values for individual transformed, oncogenic cell lines.
p. 19 **References for Supporting Information Figures 1-9**

## Experimental materials & methods section

**Safety statement**

No unexpected or unusually high safety hazards were encountered for the described experimental procedures.

**Cell lines and materials**

HEK293T cells were purchased from the UCSF cell culture facility, A549 was gifted by the Rosenberg lab at UCSF, isogenic MCF10A cell lines were constructed previously,[3,9] and PL5 were a gift from the laboratory of E. Scott Seeley (Stanford University, Stanford, California, USA) as described previously[10]. Primary human T-cells were isolated from leukoreduction chamber residuals following Trima Apheresis (Blood Centers of the Pacific, San Francisco, CA) using established protocols[11], stimulated with soluble ImmunoCult™ CD3/CD28 Activator (STEMCELL), and expanded for 14 days in ImmunoCult™-XF T Cell Expansion Medium (STEMCELL) supplemented with 500 U/mL IL-2 (GoldBio). Cell cultures were maintained in ATCC recommended conditions. All cells were cultured at 37 ˚C in a 5% $CO_2$ humidified incubator and passaged no more than 15 times. Cells were tested for mycoplasm contamination yearly.

Biotinylated peptide ester was prepared by solid phase peptide synthesis as previously described[12].The following reagents were purchased from Sigma Aldrich unless otherwise noted: O-[9-(aminooxy)nonyl]hydroxylamine, 5,5-dithio-bis-(2-nitrobenzoic acid) (Thermo Scientific, #22585), Bis-aminooxy-PEG2 (BroadPharm, BP-23592), Bis-aminooxy-PEG7 (Broad Pharm BP-23591), sodium periodate, aniline, iodoacetamide.

**N-terminal mutagenesis and *B. subtilis* transformation with a prodomain-A1S-stabiligase**

A site-directed mutagenesis reaction mixture was prepared with the forward primer (5'-GATCACGTAGCACATGCGTACCCGTGCCTTACGGCGTATCAC-3') and the reverse primer (5'-GTGATACGCCGTAAGGCACGGGTACGCATGTGCTACGTGATC-3') at 0.5 µM each, the plasmid prep-pro-stabiligase-hexahistidine(100 ng),[12] dNTPs (0.2 mM), 2.5 mM $MgSO_4$, 1X KOD hot start DNA polymerase buffer, and KOD HOT Start DNA polymerase (0.02 U/µl). After thermocycle amplification, the reaction mixture was digested with Dpn1 (0.8 U/µl) at 1 hr, 37 ˚C at 220 rpm. The PCR product was then added to *E. coli* XL10 for transformation. The stabiligase(A1S) plasmid was then transformed into *E. coli* ER1821, concatemeric DNA was purified, and used to transform *B. subtilis* BG2864 using previous methods.[8] Transformed bacteria was then plated on Luria Bertani agar containing 10 µg/ml chloramphenicol.

**Expression and purification of stabiligase(A1S)**

An overnight culture of *B. subtilis* BG2864 transformed with stabiligase(A1S) was grown in Luria Bertani (LB) broth supplemented with 5 µg/ml chloramphenicol. The culture was then diluted 1:100 into 200 ml of 2XYT broth in a 1 L baffled flask. 1 mM $CaCl_2$ and 10 µg/ml chloramphenicol was added to the media, and cells grew at 37 ˚C, 280 rpm for 18-20 hours. Supernatant was clarified (4000x*g*, 15 min, 4 ˚C) and a 3:1 v/v of cold EtOH was added. Precipitated material was collected by centrifugation (4000x*g*, 15 min, 4 ˚C), and protein was resolubilized in 40 ml of buffer A (50 mM sodium phosphate (pH 8), 300 mM NaCl, and 5 mM imidazole). After removing insoluble debris (4,000x*g*, 15 minutes, 4 ˚C), clarified solution was incubated with nickel resin pre-equilibrated in buffer A for 1 hour at 4˚C. Unbound material was removed by gravity filtration and resin was washed consecutively (2X) with buffer A provided at

eight times the resin volume, and then a final addition of buffer A + 20 mM imidazole. Buffer A + 400 mM imidazole was then added to the resin to elute protein. Intact protein mass of stabiligase(A1S) was determined by intact protein mass spectrometry (MS) using a Xevo G2-XS Mass Spectometer (Waters) equipped with a LockSpray (ESI) source and a Acquity Protein BEH C4 column (2.1 mm inner diameter, 50 mm length, 300 Å pore size, 1.7 μm particle size) connected to an Aquity I-class liquid chromatography system (Waters). Deconvolution of mass spectra was performed using the maximum entropy (MaxEnt) algorithm in MassLynx 4.1 (Waters). Protein concentration was determined by nanodrop and if required, concentrated using an amicon 10K MWCO tube further to 4-6 mg/ml prior to N-terminal conjugation.

**Stabiligase N-terminal conjugation**

Solid 5,5-dithio-bis-(2-ntirobenzoic acid) (2 mM, DNTB, Ellman's reagent; Sigma Aldrich) was added to nickel eluate for a 30 min incubation at room temperature. TNB- Cys221 conversion was monitored by intact protein MS and following complete thiol-protection, excess DNTB was removed using a disposable P10-desalt column (Cytiva, Catalog # 17085101). Protein was eluted into 50 mM sodium phosphate (pH 8), 300 mM NaCl. 5X molar excess of sodium periodate (100 mM in $dH_2O$; Sigma Aldrich) was added for a 5 min incubation at 4 ˚C. Complete N-terminal oxidation was confirmed using intact protein MS, and excess sodium periodate was removed using a P10-desalt column with buffer B (50 mM sodium phosphate (pH 6.5), 300 mM NaCl) for elution. For imine ligation, stabiligase (2-6 mg/ml) was incubated with 80-100 molar excess of either adipic acid or O-[9-(aminooxy)nonyl]hydroxylamine (Sigma Aldrich) and aniline (10 mM) in buffer B. After an overnight incubation at 4 ˚C, intact protein MS monitoring after 12-16 hours. Desalted, conjugated stabiligase was then buffer exchanged into buffer C (50 mM sodium phosphate (pH 7.4), 300 mM NaCl using a P-10 desalting column. Lastly, 50 mM TCEP (500 mM, 1M Tris (pH 8.5); solid from Sigma Aldrich, #75259) was added for a 20-minute incubation. The resulting deprotected, conjugated stabiligase was analyzed using intact protein MS, desalted using a P10 column, and exchanged into buffer C. Stabiligase was then purified on S75 10/300 GL column or HiLoad 16/600 Superdex 75 pg (Cytiva) equilibrated with buffer C and fractions corresponding to monomeric conjugated-stabiligase were collected. Single-use aliquots (150-200 μM conjugated stabiligase) were flash-frozen in liquid nitrogen and then stored at -80 ˚C.

**General protocol for cell surface stabiligase tethering and N-terminal labeling protocol for flow cytometric analysis and western blot detection**

HEK293T cells seeded in T75 $cm^3$ plates were incubated at 37 ˚C in 5% $CO_2$. Confluent cells were gently washed with DBPS and then incubated with PBS with 0.04% EDTA, free of $Ca^{2+}/Mg^{2+}$ for 10 minutes. Dissociated cells were collected, pelleted (400x*g,* 5 min, 4 ˚C), washed DPBS (pH 7.4), re-pelleted, and then transferred to 1.5 mL Eppendorf tubes with DPBS (pH 7.5). Cells were treated with 500 μM sodium periodate (stock solution of 100 mM sodium periodate in $dH_2O$) and gently rocked for 15 min at 4 ˚C. Cells were re-pelleted (400x*g*, 5 min, 4 ˚C), washed with cold DPBS (pH 6.5), re-pelleted, and resuspended in cold DPBS (pH 6.5) to which 10 mM aniline and then 5 μM functionalized stabiligase was added. Cells were then gently rotated at 4 ˚C for 10 min, re-pelleted, washed with DPBS (pH 7.5), re-pelleted, and then resuspended in stabiligase-reaction mixture consisting of 50 mM Tricine (pH 8), 150 mM NaCl, and then 1% DMSO for a final 1 mM concentration of the biotinylated peptide ester (200 mM, DMSO). Cells were then gently rotated for 10 min at room temperature before pelleting cells (400xg, 5 min, 4

˚C), washing cells with DPBS (pH 7.4), and then re-pelleting. For flow cytometry analysis of tethering and ligation, cells were stained with AlexaFluor647-anti-histidine (1:2000) and AlexaFluor488-streptavidin (1:5000) in DPBS (pH 7.4) with 3% BSA. Cells were pelleted and washed in DPBS with 3% BSA prior to analysis. Samples were analyzed on a Beckman Coulter CytoFlex flow cytometry and then using FlowJo software.

For western blot evaluation of N-terminal ligation, pelleted cells were fractionated using a subcellular fractionation kit (Thermo Fisher, # 78840) according to the manufacturer's instructions for both the cytoplasmic extraction and the membrane extraction. Protein concentration was measured by Pierce BCA (Thermo Fisher, #23225) and fraction aliquots were frozen at -20˚C before use. 15 µg of protein from each fraction was subjected to SDS-PAGE and blotted on PVDF membranes using an iBlot transfer stack (Thermo Fisher, #IB301002). Biotinylation was detected using IRDye 800CW Streptavidin (1:5000 dilution; LI-COR Biosciences, #926-32230) and total protein loaded in each lane was assessed using REVERT protein staining kit (LI-COR Biosciences, #926-11015) according to the manufacturer's instructions. Near-infrared (NIR) western blot images were analyzed using an Odyssey Li-COR imaging system and further analyzed using ImageJ.

**General protocol for cell surface N-terminomics**

For adherent cells (HEK293T, MCF10A, PL5, A549), cells were cultured to approximately 90% confluency in plates that yielded 7-12x$10^6$ cells. For SILAC-cultured MCF10A cells were passaged at least eight times prior in the light and heavy-isotopic media. Cells were harvested by washing plates with cold DPBS and incubating with PBS with 0.04% EDTA, free of $Ca^{2+}$/$Mg^{2+}$. For SILAC-based experiments, cells were combined in equal numbers after cell counting using a Bio-rad TC20 automatic cell counter. Cells were pelleted by centrifugation (400xg, 5 min, 4˚C), washed with 1 mL DPBS (pH 6.4) and re-pelleted before proceeding further. For primary immune cells ($CD4^+$ T cells, $CD8^+$ T cells, NK), cells were counted using a hemocytometer and 20-30x$10^6$ cells were collected, pelleted by centrifugation (400xg, 5 min, 4˚C), washed with DPBS (pH 7.4) and pelleted before proceeding further.

After harvesting cells, all cell types were treated similarly. Cells were resuspended in 1 mL DPBS (pH 6.4) containing 500 µM $NaIO_4$ (100 mM $NaIO_4$ stock solution in $dH_2O$) in a low-bind Axygen 1.7 mL tube (Thermo Fisher). The sodium periodate reaction mixture was then covered from light and rotated on ice at 300 rpm for 10 minutes. Cells were re-pelleted by centrifugation (400xg, 5 min, 4 ˚C), washed with DPBS (pH 7.4), and then resuspended in DPBS (pH 6.4). To a final reaction volume of 1 ml, 10 mM aniline (Sigma Aldrich) and then 5 µM GT-stabiligase was added and the oxime-ligation proceeded on a rotating platform covered from light. Cells were re-pelleted by centrifugation (400x*g*, 5 min, 4˚C), washed with 1 mL DPBS (pH 7.4), and then resuspended in the subtiligase master mix (100 mM Tricine (pH 8), 150 mM NaCl, 1 mM biotinylated peptide ester (1% final DMSO). Cells were gently rocked at room temperature for 15 minutes before centrifugation at 400xg, 5 min, 4 ˚C. Cells were washed two times with DPBS (pH 7.4) and then flash-frozen in liquid nitrogen for storage at -80 ˚C prior to mass spectrometry sample work-up.

**Sample preparation for LC-MS-MS analysis**

For sample processing, frozen cell pellets were thawed on ice and then resuspended in 1 mL lysis buffer (RIPA, Radioimmunoprecipitation assay buffer) supplemented with a complete protease inhibitor cocktail (Roche) and DNase 1 (100 µg/ml). After a 30 min incubation at 16 ˚C,

cells were briefly sonicated (3 pulses, 20% amplitude) and cell debris was removed by centrifugation (20,000x*g* for 10 min, 4 ˚C). Clarified supernatant was added to 250 µl NeutrAvidin agarose beads (Pierce, #29200) and incubated for 3 hours at 16 ˚C. Non-specific bound proteins were removed by collecting beads with spin columns (Pierce, #69725) and washing iteratively with 5 x 1 mL of RIPA buffer, 5 x 1 mL 100 mM Tris (pH 7.5) and 1 M NaCl, 5 x 1 mL 50 mM ammonium bicarbonate and 2 M Urea. Beads were then transferred to 1.5 mL maximum recovery tubes (Axygen, MCT-150-L-C; 1 mL of 50 mM ammonium bicarbonate and 2 M Urea). TCEP (5 mM) was added to the resuspended mixture and then samples were incubated 55 ˚C for 30 minutes at 800 rpm. After samples were equilibrated to rt, the iodoacetamide (14 mM) was added and incubated for 30 minutes. Beads were centrifuged (800x*g*, 1 min) and washed two times with 50 mM ammonium bicarbonate with 2 mM Urea. 6 µg of sequencing grade trypsin (Promega, #V5113) was added for an overnight incubation at rt. Beads were collected and washed as described above prior to trypsin digestion. The collected beads were then washed with an additional 3 x 1 mL d$H_2O$ and then resuspended in TEV buffer (700 µl, 50 mM Tris (pH 8.0), 0.5 mM EDTA, 1 mM dithiothreitol). 8 µg of TEV protease was added to each sample and the beads were incubated at rt overnight. Beads were removed with spin columns, and the supernatant was collected into a fresh 1.5 mL maximum recovery tube. Beads were washed with 2 x 100 µl of d$H_2O$. The collected supernatant was dried to completion using a genovac drying system and then desalted using Preomics iST desalting columns per the manufacturer's instructions.

**Liquid chromatography mass spectrometry analysis of N-termini peptides**

Desalted peptides (200 ng) were loaded onto a timsTOF Pro equipped with a CaptiveSpray source and a nanoElute line (Bruker; Hamburg, Germany). The peptides were separated on a 25 cm, ReproSil c18 1.5 µM 100 A column (PepSep, PN. # PSC-25-150-15-UHP-nc) using a step-wise linear gradient method with $H_2O$ in 0.1% Formic acid and acetronitrile with 0.1% formic acid (solvent B): 5-30% solvent B for 90 min at 0.5 µl/min, 30-35% solvent B for 10 min at 0.6 µl/min, 35-95% solvent B for 4 min at 0.5 µl/min, 95% hold for 4 min at 0.5 µl/min). Acquired data was collected in a data-dependent acquisition mode with ion mobility activated in PASEF mode. MS and MS/MS spectra were collected with m/z ranging from 100 to 1700 in positive mode.

All acquired data was searched using PEAKS online Xpro 1.6 (Bioinformatics Solutions Inc.; Ontario, Canada).[13] Spectral searches were performed using a custom FASTA-formatted dataset containing Swissprot-reviewed human proteome file with gene ontology localized the plasma membrane (downloaded from Uniprot knowledge database, #entries)[5]. A precursor mass error tolerance was set to 20 ppm and a fragment mass error tolerance was set at 0.03 ppm. Peptides, ranging from 6 to 45 amino acids in length, were searched in semi-specific tryptic digest mode with a maximum of two missed cleavages. Carbidomethylation (+57.0214 Da) on cysteines was set as a static modification and methionine oxidation (+15.994) was set as a variable modification. N-terminal specific peptides were identified by the N-terminal amino-butyric mass tag (+85.052764). Lastly, peptides were filtered based on a false discovery rate (FDR) of 1%.

SILAC-labeled datasets were also analyzed in PEAKS online Xpro 1.6 with the following alterations. Two additional variable modifications were set to account for the isotopic-labels: 13C(6)15N(2) and 13C(6) 15N(4), quantified peptides were matched between experimental replicates and enrichments were normalized based on total ion chromatograph (TIC). Output PEAKS files contained SILAC peptide ratios. If peptides overlapped with the same N-terminal site (*i.e.*, tryptic C-termini, different oxidation), peptides were grouped together and the average ratio was reported for a given experimental dataset. Across replicates, peptides were removed if

observed with high variation (coefficient of variation>0.7) and peptides were quantified if present in two biological replicates. The final N-terminal peptide ratio was then reported as the median $log_2$ fold-change value alongside the shortest N-terminal tryptic peptide sequence.

N-terminomics data output from PEAKS online XPro 1.6 was further analyzed using custom scripts available on Github (https://github.com/krschaefer/GTNTerm-inomics and https://github.com/crystaljie/surface_proteolysis_analysis.git). All mass spectrometry data are available via PRIDE with the identifier PXD033811.

**Analysis of N-terminomics datasets**

With custom python scripts and the Swiss-prot reviewed human proteome exported from the Uniprot knowledge database, MS files processed in PEAKS were further processed to filter neo-N-termini according to the following features: topological domain of peptide, type of membrane protein, subcellular localization, predicted glycosites, distance to signal sequence sites, distance to pro-peptide sequence sites (https://github.com/krschaefer/GTNTerminomics). Cell surface displayed proteins were annotated if identified by the following criteria: extracellular topology of membrane proteins (single-pass or multi-pass); proteins with a subcellular localization as extracellular secreted proteins; or GPI-linked proteins localized to the plasma membrane. For the location of the following proteolytic cleavages--initiator methionine removal, signal peptide cleavage, propeptide cleavages—a precision ruler set within 10 amino acids from the annotated site was used. Additionally, neo-N-termini were paired to cell surface capture surfaceomics data from the previous reference[3] and the cell surface atlas public datasets (https://wlab.ethz.ch/cspa/#downloads).[6] Additional analyses included amino acid distances from neo-N-termini to the most proximal transmembrane helix or GPI-linked anchor and neo-N-termini relative to annotated domain architectures. In some instances, incomplete annotations (missing topological domain orientations or single-pass membrane proteins lacking typology annotations) were observed and topology of N-termini were manually annotated based on the Protein Protter server (http://wlab.ethz.ch/protter/start/).[6] Gene ontology enrichments were obtained using the webgestalt server using an FDR cut-off at 1%[4], and functional protein assignments were made using the Panther server.[14]

For a topfind 4.1 (https://topfind.clip.msl.ubc.ca) comparison,[15] protein accessions and peptide sequences were submitted as lists with default parameters and a precision range within 10 amino acids. For sequence logo generation, IceLogo was used with a precompiled Swiss-Prot *Homo sapiens* composition as the reference set.

Secondary structures and relative solvent accessibility for the identified cleavage sites (P4-P4') were analyzed using an in-house informatics pipeline written in R (https://github.com/crystaljie/surface_proteolysis_analysis.git). In brief, DSSP algorithm was used to obtain the exact secondary structure information or relative solvent accessibility based on PDB or AlphaFold 2.0 structures.[5,7,16]

**Western blot analysis of proteolytic substrates**

For validating quantitative ratios of enriched neo-N-termini, MCF10A cells (empty vector (ev), *her2*, *krasG12V*) were grown in 10 $cm^3$ plates to 90% confluency. Cells were harvested by washing plates with cold DPBS and incubating with PBS with 0.04% EDTA, free of $Ca^{2+}/Mg^{2+}$ incubation. Cells were pelleted, washed three times with DPBS, and then resuspended in RIPA buffer supplemented with complete protease inhibitor cocktail (Roche) and DNase 1 (100 µg/ml).

After a 30 min incubation at 16 ˚C, cell debris was removed by centrifugation (20,000x*g* for 10 min, 4 ˚C). Total protein concentration was determined using a Pierce BCA Protein Assay kit (# 23225) and 20 µg of protein was incubated with 4X-SDS loading buffer containing 5% ß-mercaptoethanol on ice for thirty minutes. Samples were separated using a 4-12% Bolt, Bis-Tris, invitrogen gel and then transferred to a PVDF membrane. The membrane was blocked with TBS Intercept blocking buffer (LI-COR, #927-60001) rotating for 1 hour at room temperature. The following primary antibody solutions were added for the respective protein targets: anti-Notch2 (1:1000 dilution; Cell Signaling, #5372), anti-LDLR (1:1000 dilution, R&D, # AF2148); anti-DSG-2 (1:1000 dilution, Abcam, #ab226258), Anti-T-cadherin (1:2000, CDH13; EMD Millipore, #ABT121). All primary antibody solutions also contained anti-actin (anti-mouse or anti-rabbit; 1:2000 dilution; Cell Signaling, #3700 or #4970). All with the exception of anti-DSG-2 were rocked overnight at 4 ˚C. For DSG-2, the blot was incubated with anti-DSG-2 solution for 1 hour at room temperature. After iterative washing three times with TBS-T, blots for detecting Notch2, DSG-2, T-cadherin were incubated with either IRDye-680/800-anti-mouse and IRDye-680/800 anti-rabbit (1:10000 dilution) based on the primary antibody used. For blots detecting LDLR, IRDye-800-anti-goat (1:10000 dilution) and IRDye-680-anti-mouse were used. Blots were rocked at room temperature for 1 hour in the dark before iterative washing three times with TBS-T. Near-infrared (NIR) western blot images were analyzed using an Odyssey Li-COR imaging system and further analyzed using ImageJ.

## Experimental materials and methods references:


1. Leung, K. K. *et al.* Broad and thematic remodeling of the surfaceome and glycoproteome on isogenic cells transformed with driving proliferative oncogenes. *Proc. Natl. Acad. Sci. USA* **117**, 7764-7775, doi:10.1073/pnas.1917947117 (2020).
2. Martinko, A. J. *et al.* Targeting RAS-driven human cancer cells with antibodies to upregulated and essential cell-surface proteins. *Elife* **7**, doi:10.7554/eLife.31098 (2018).
3. Lim, S. A. *et al.* Targeting a proteolytic neoepitope on CUB domain containing protein 1 (CDCP1) for RAS-driven cancers. *J. Clin. Invest.* **132**, doi:10.1172/JCI154604 (2022).
4. Byrnes, J. R. *et al.* Hypoxia is a dominant remodeler of the effector T cell surface proteome relative to activation and regulatory T cell suppression. *Mol. Cell Proteomics*, 100217, doi:10.1016/j.mcpro.2022.100217 (2022).
5. Weeks, A. M., Byrnes, J. R., Lui, I. & Wells, J. A. Mapping proteolytic neo-N termini at the surface of living cells. *Proc. Natl. Acad. Sci. USA* **118**, doi:10.1073/pnas.2018809118 (2021).
6. Weeks, A. M. & Wells, J. A. Engineering peptide ligase specificity by proteomic identification of ligation sites. *Nat. Chem. Biol.* **14**, 50-57, doi:10.1038/nchembio.2521 (2018).
7. Tran, N. H. *et al.* Deep learning enables de novo peptide sequencing from data-independent-acquisition mass spectrometry. *Nat. Methods* **16**, 63-66, doi:10.1038/s41592-018-0260-3 (2019).
8. The UniProt Consortium. UniProt: the universal protein knowledgebase. *Nucleic Acids Research* **45**, D158-D169, doi:10.1093/nar/gkw1099 (2016).
9. Bausch-Fluck, D. *et al.* A mass spectrometric-derived cell surface protein atlas. *PLoS One* **10**, e0121314, doi:10.1371/journal.pone.0121314 (2015).
10. Liao, Y., Wang, J., Jaehnig, E. J., Shi, Z. & Zhang, B. WebGestalt 2019: gene set analysis toolkit with revamped UIs and APIs. *Nucleic Acids Res.* **47**, W199-W205, doi:10.1093/nar/gkz401 (2019).
11. Mi, H., Muruganujan, A. & Thomas, P. D. PANTHER in 2013: modeling the evolution of gene function, and other gene attributes, in the context of phylogenetic trees. *Nucleic Acids Res.* **41**, D377-386, doi:10.1093/nar/gks1118 (2013).
12. Fortelny, N., Yang, S., Pavlidis, P., Lange, P. F. & Overall, C. M. Proteome TopFIND 3.0 with TopFINDer and PathFINDer: database and analysis tools for the association of protein termini to pre- and post-translational events. *Nucleic Acids Res* **43**, D290-297, doi:10.1093/nar/gku1012 (2015).

13. Zhou, J. *et al.* Deep profiling of protease substrate specificity enabled by dual random and scanned human proteome substrate phage libraries. *Proc. Natl. Acad. Sci. USA* **117**, 25464-25475, doi:10.1073/pnas.2009279117 (2020).
14. Varadi, M. *et al.* AlphaFold Protein Structure Database: massively expanding the structural coverage of protein-sequence space with high-accuracy models. *Nucleic Acids Res.* **50**, D439-D444, doi:10.1093/nar/gkab1061 (2022).

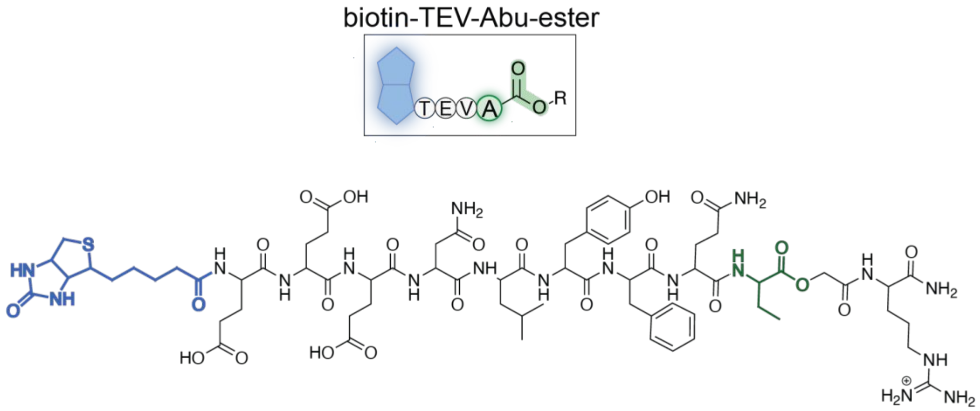


**Figure S1.** Chemical structure of the biotinylated peptide ester substrate transferred to N-terminal amines in the presence of stabiligase. Featuring a biotin handle (blue), a TEV-protease cleavage sequence, and an Amino-butyric acid (Abu, green) mass tag, the peptide ester was prepared using solid-phase peptide synthesis as reported previously.[8]

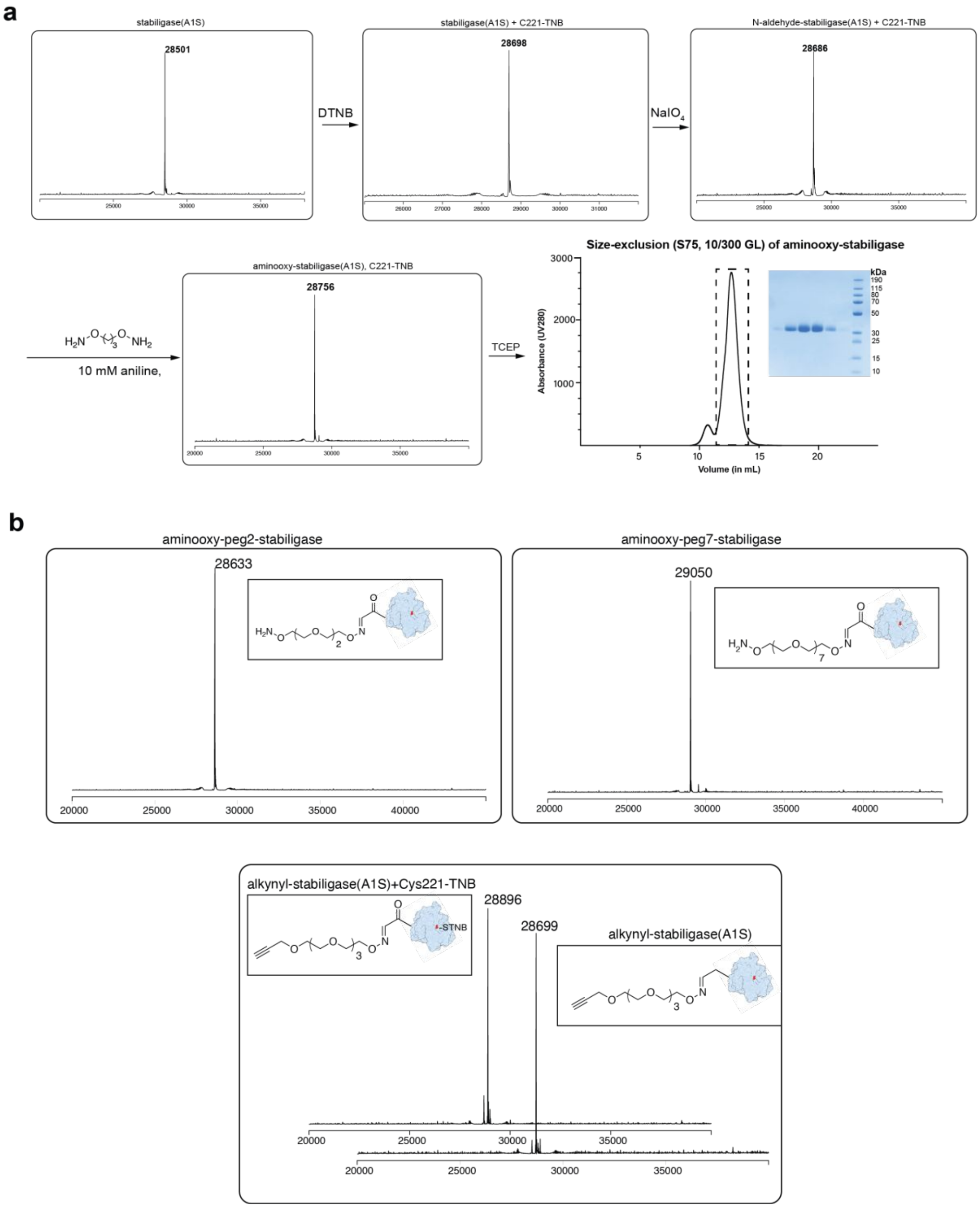


**Figure S2.** Scheme depicting the site-selective conjugation of stabiligase (A1S) and intact protein mass spectrometry traces for various synthetic steps and conjugated stabligases. a) Stabiligase(A1S) was purified using similar conditions as reported.[8] Purified enzyme was then treated with Ellman's reagent (5,5-dithio-bis-(2-nitrobenzoic acid), DTNB) to create a TNB-C221 adduct (30 min, rt). A brief sodium periodate incubation (5 min, 4˚C) created an N-terminal aldehyde. In the presence of nucleophilic reagents (shown here, O-[9(aminooxy)nonyl]-hydroxylamine) and aniline (overnight, 4˚C), the N-terminus of stabiligase was fully conjugated. Aminooxy-modified stabiligase was treated with TCEP to remove the TNB-group (20 min, rt), and the enzyme was further purified using size-exclusion chromatography. **b)** Deconvoluted intact mass spectrometry traces represent additional functionalized-stabiligases prepared for this study.

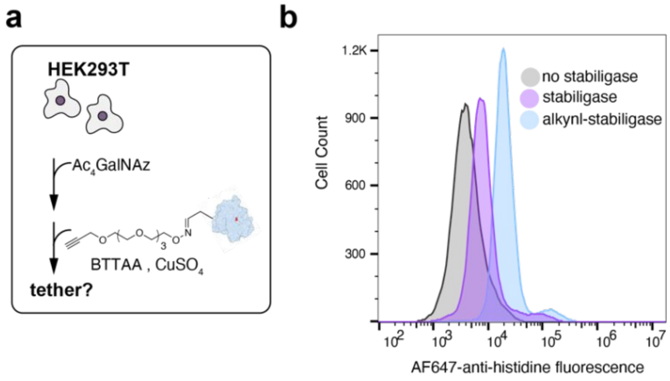


**Figure S3.** Click-reaction conditions provide modest alkynyl-stabiligase tethering to metabolically-labeled glycans. Cells were cultured with peracetylated-GalNAz similar to previous literature.[1,2] After 48 hours, cells were harvested, washed three times, and then incubated with N-terminal-alkynyl-stabiligase and additional reagents required for a copper-based click reaction on living cells.[2] HEK293T cells were subsequently washed and tethering was determined by flow cytometry with AlexaFluor647-anti-histidine. Modest levels of stabiligase attachment to cells was observed using this strategy (for comparison also see main text Figure 2).

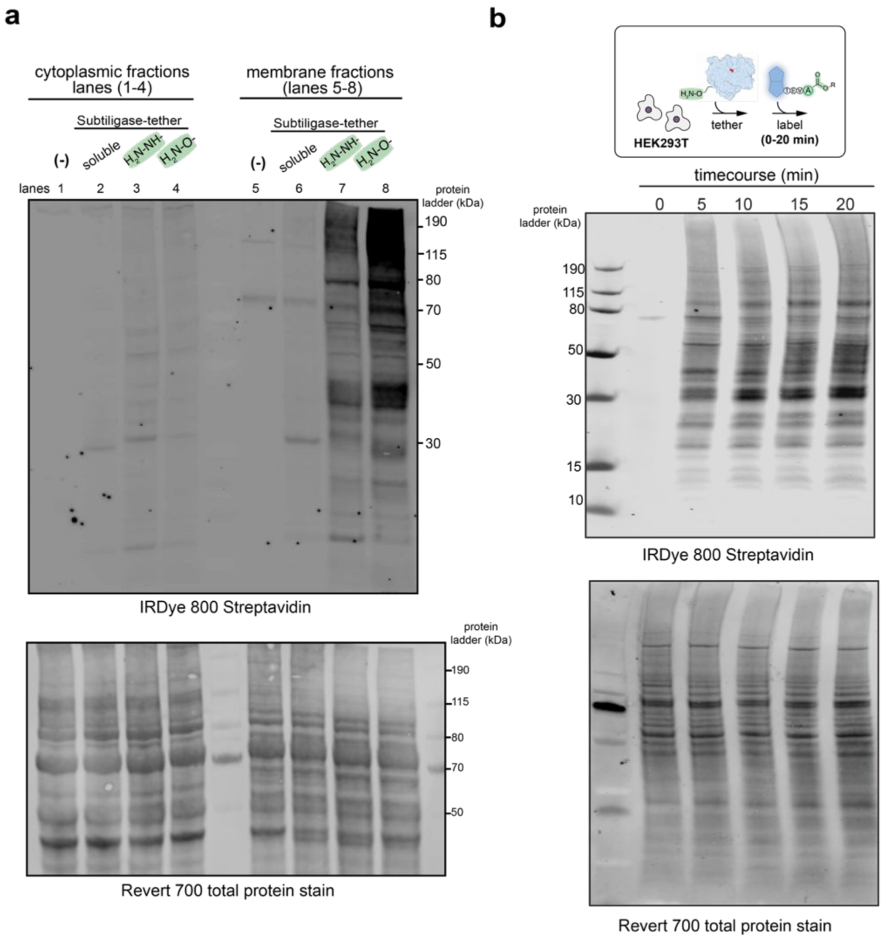


**Figure S4.** Glycan-tethered stabiligase broadly labels membrane proteins. a) Under tethering conditions described in the main text, HEK293T cells attached to GT-stabiligase were then incubated briefly with the biotinylated peptide ester substrate (15 min, rt). Cells were then lysed and fractionated using subcellular fractionation (Thermo Fisher, # 78840). 20 µg of cytoplasmic and membrane fractions were loaded onto a SDS-PAGE gel, transferred to PVDF, and then blotted with Revert 700 total protein stain (LI-COR) and IRDye-800-Streptavidin (LI-COR). b) HEK293T cells tethered to aminooxy-stabiligase were treated the biotinylated peptide ester for 5 min intervals (0-20 min). For western blot detection, samples were prepared as described in a). These experiments were performed on at least three individual times with similar results.

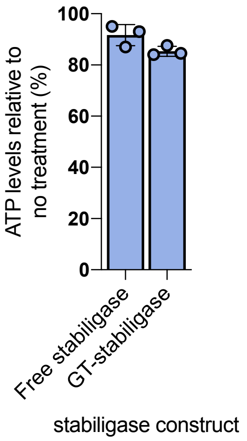


**Figure S5.** GT-stabiligase tethering and N-terminal labeling does not significantly affect cytotoxicity. HEK293T cells were treated with sodium periodate, and then incubated with either soluble stabiligase (5 μM) or aminooxy-stabiligase under oxime-ligation conditions as described in the main text. The biotinylated peptide ester was added to cells (15 min, rt) and for reactions with soluble stabiligase, an additional 5 μM soluble stabiligase was added. Cell viability was evaluated using the CellTiter Glo luminescence assay according to the manufacturer's instructions to quantify ATP levels, and luminescence for stabiligase reactions was normalized relative to untreated HEK293T cells as a control. Independent experiments were performed three times, and the mean ± S.D. was reported.

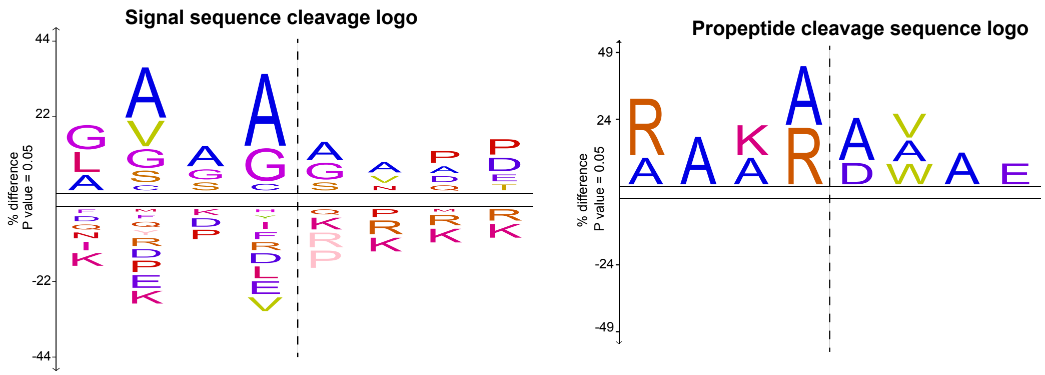


**Figure S6.** Icelogos (P4-P4') for N-termini located at signal peptide- or propeptide- cleavage junctions that were identified using cell surface N-terminomics.

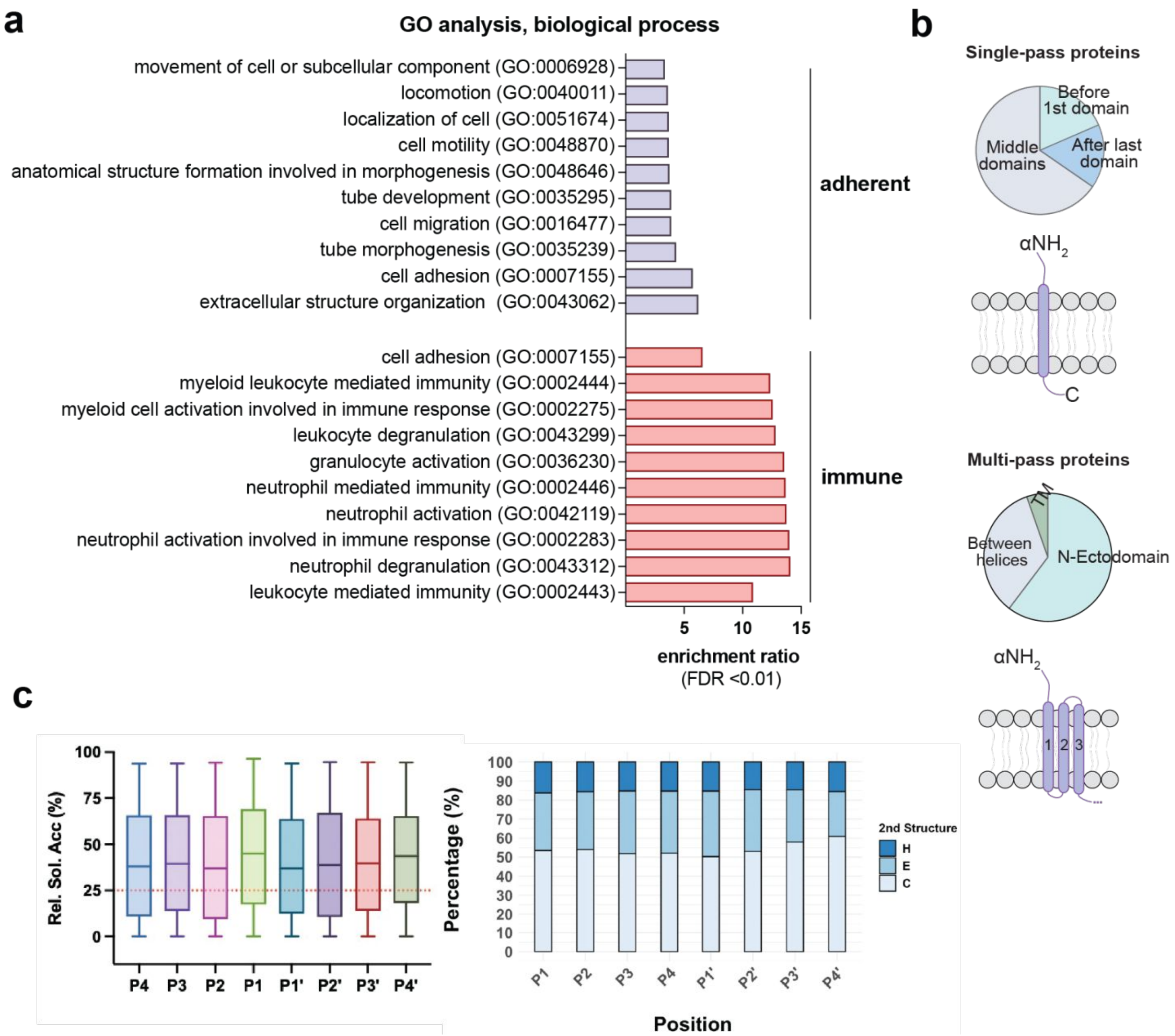


**Figure S7.** Further characterization of extracellular N-termini identified from cell surface N-terminomics across different cellular contexts. a) Gene ontology analysis (GO) of biological processes for neo-N-termini localized to extracellular domains beyond maturation cleavages for adherent cells and immune cell types.[4] b) Localization of extracellular cleavage sites relative to domains for single-pass membrane proteins and regions between multi-pass transmembrane helices.[5,6] c) Using PDB deposited structures and structural predictions made by AlphaFold 2.0,[7] predicted secondary structures and surface accessibility of the extracellular cleavage sites were made. For secondary structure assignment, H represents helix, E represents sheets, C represents loops. Detailed descriptions of these analyses are available in Github (https://github.com/crystaljie/surface_proteolysis_analysis.git). Also see Table S4.

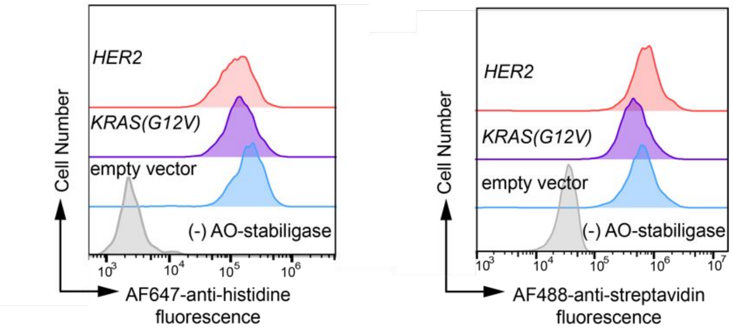


**Figure S8.** Flow cytometry analysis demonstrates that GT-stabiligase attachment and N-terminal ligation is not affected by oncogene-driven physiological changes. MCF10A cell lines transformed with *HER2*, *KRAS(G12V)*, or a control empty vector (*ev*) were tethered with GT-stabiligase and treated with the biotinylated peptide ester substrate as described in the main text. Flow cytometry analysis shows similar stabiligase tethering (monitored by AlexaFluor647-anti-histidine fluorescence) and ligation activity (monitored by AlexaFluor488-streptavidin fluorescence) for all three MCF10A transformed cell lines.

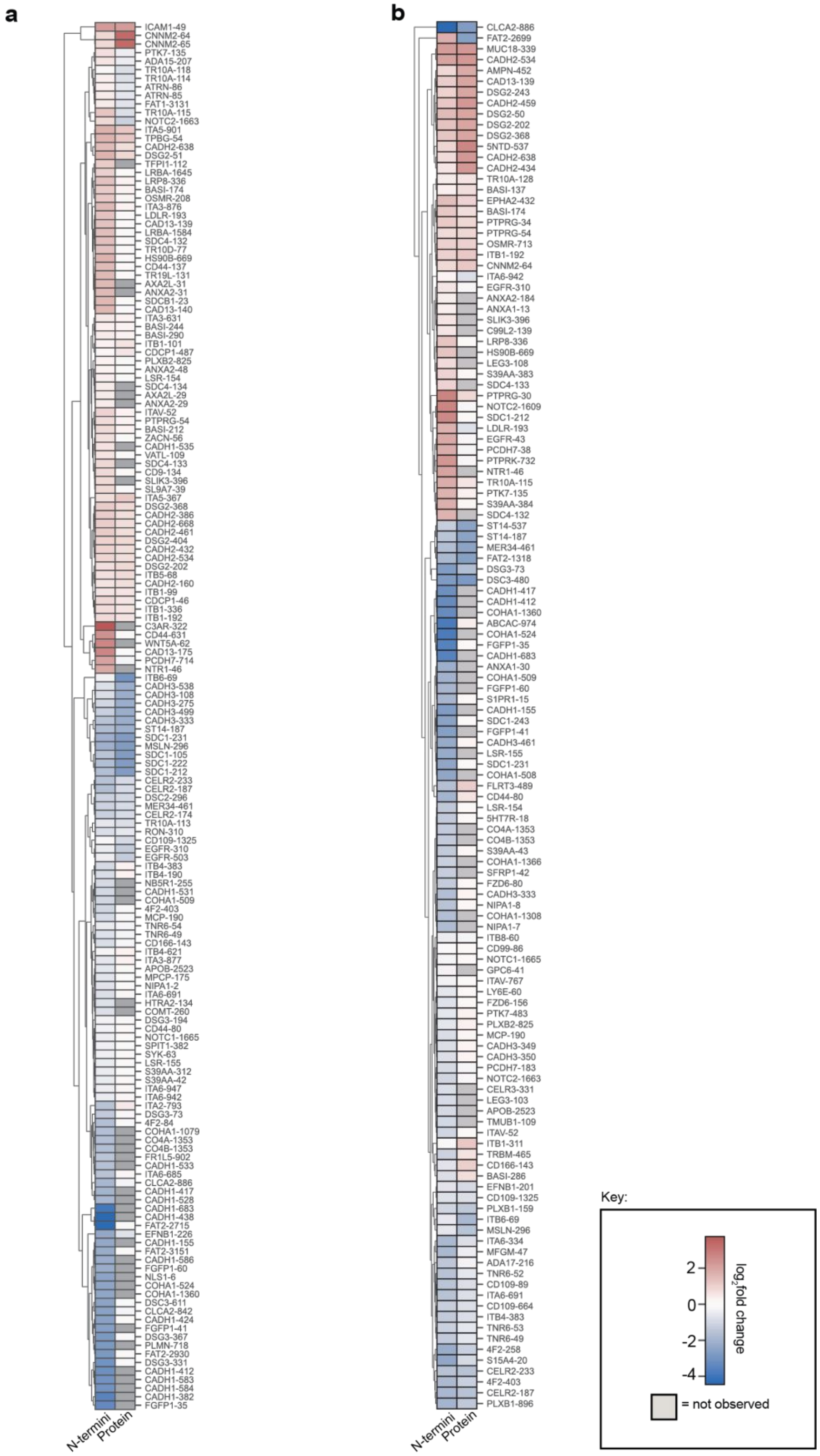


**Figure S9.** Comparisons between neo-N-termini fold changes and protein abundance changes for single oncogene-driven datasets shows modest correlation. Heat maps represent fold-changes (1.8 or higher threshold) in the presence of the oncogenes *HER2* for panel a) or *KRAS(G12V)* for panel b). The protein and N-termini peptide start position are annotated for each row. For proteins not observed in CSC proteomics,[3] the protein abundance row is colored gray to indicate a missing value.

## References for Supplementary Figures 1-9:


1. Debets, M. F. *et al.* Metabolic precision labeling enables selective probing of O-linked N-acetylgalactosamine glycosylation. *Proc. Natl. Acad. Sci. USA* **117**, 25293-25301, doi:10.1073/pnas.2007297117 (2020).
2. Hong, V., Steinmetz, N. F., Manchester, M. & Finn, M. G. Labeling live cells by copper-catalyzed alkyne--azide click chemistry. *Bioconjug. Chem.* **21**, 1912-1916, doi:10.1021/bc100272z (2010).
3. Leung, K. K. *et al.* Broad and thematic remodeling of the surfaceome and glycoproteome on isogenic cells transformed with driving proliferative oncogenes. *Proc. Natl. Acad. Sci. USA* **117**, 7764-7775, doi:10.1073/pnas.1917947117 (2020).
4. Liao, Y., Wang, J., Jaehnig, E. J., Shi, Z. & Zhang, B. WebGestalt 2019: gene set analysis toolkit with revamped UIs and APIs. *Nucleic Acids Res.* **47**, W199-W205, doi:10.1093/nar/gkz401 (2019).
5. The UniProt Consortium. UniProt: the universal protein knowledgebase. *Nucleic Acids Research* **45**, D158-D169, doi:10.1093/nar/gkw1099 (2016).
6. Bausch-Fluck, D. *et al.* A mass spectrometric-derived cell surface protein atlas. *PLoS One* **10**, e0121314, doi:10.1371/journal.pone.0121314 (2015).
7. Varadi, M. *et al.* AlphaFold Protein Structure Database: massively expanding the structural coverage of protein-sequence space with high-accuracy models. *Nucleic Acids Res.* **50**, D439-D444, doi:10.1093/nar/gkab1061 (2022).
8. Weeks, A. M. & Wells, J. A. Engineering peptide ligase specificity by proteomic identification of ligation sites. *Nat. Chem. Biol.* **14**, 50-57, doi:10.1038/nchembio.2521 (2018).
9. Martinko, A. J. *et al.* Targeting RAS-driven human cancer cells with antibodies to upregulated and essential cell-surface proteins. *Elife* **7**, doi:10.7554/eLife.31098 (2018).
10. Lim, S. A. *et al.* Targeting a proteolytic neoepitope on CUB domain containing protein 1 (CDCP1) for RAS-driven cancers. *J. Clin. Invest.* **132**, doi:10.1172/JCI154604 (2022).
11. Byrnes, J. R. *et al.* Hypoxia is a dominant remodeler of the effector T cell surface proteome relative to activation and regulatory T cell suppression. *Mol. Cell Proteomics*, 100217, doi:10.1016/j.mcpro.2022.100217 (2022).
12. Weeks, A. M., Byrnes, J. R., Lui, I. & Wells, J. A. Mapping proteolytic neo-N termini at the surface of living cells. *Proc. Natl. Acad. Sci. USA* **118**, doi:10.1073/pnas.2018809118 (2021).
13. Tran, N. H. *et al.* Deep learning enables de novo peptide sequencing from data-independent-acquisition mass spectrometry. *Nat. Methods* **16**, 63-66, doi:10.1038/s41592-018-0260-3 (2019).
14. Mi, H., Muruganujan, A. & Thomas, P. D. PANTHER in 2013: modeling the evolution of gene function, and other gene attributes, in the context of phylogenetic trees. *Nucleic Acids Res.* **41**, D377-386, doi:10.1093/nar/gks1118 (2013).
15. Fortelny, N., Yang, S., Pavlidis, P., Lange, P. F. & Overall, C. M. Proteome TopFIND 3.0 with TopFINDer and PathFINDer: database and analysis tools for the association of protein termini to pre- and post-translational events. *Nucleic Acids Res.* **43**, D290-297, doi:10.1093/nar/gku1012 (2015).
16. Zhou, J. *et al.* Deep profiling of protease substrate specificity enabled by dual random and scanned human proteome substrate phage libraries. *Proc Natl Acad Sci U S A* **117**, 25464-25475, doi:10.1073/pnas.2009279117 (2020).
