# Supplementary material for: Direct Identification of Proteolytic Cleavages on Living Cells Using a Glycan-Tethered Peptide Ligase
Source: ACS Cent Sci. 2022 Oct 11;8(10):1447–56. doi: 10.1021/acscentsci.2c00899 (PMC9615116; doi:10.1021/acscentsci.2c00899)
Supplement: Supplementary file 6 — oc2c00899_si_006.pdf [file oc2c00899_si_006.pdf]

oc-2022-00899w.R1

Name: Peer Review Information for "Direct identification of proteolytic cleavages on living cells using a glycan tethered peptide ligase"

First Round of Reviewer Comments

Reviewer: 1

Comments to the Author

In this manuscript, the authors demonstrate the development of a novel tool for the labeling and subsequent identification of proteolytic cleavage sites at the cell surface. By tethering their engineered peptide ligase to extracellular glycans containing sialic acids, the authors distribute the enzyme across the cell surface, and subsequently label the N-termini of nearby proteins. After optimization, the authors demonstrate selective labeling of membrane proteins. Upon proteomic analysis, the authors identify several novel proteolytic cleavage sites across multiple biological contexts and proceed to validate the cleavage of several of these proteins across cancer cell lines with the HER2 and KRAS(G12V) mutations. The authors accomplished a well considered optimization of the N-terminomics labeling strategy. We especially note the consideration of multiple different strategies for labeling and tethering (multiple stabiligases, click-chemistry route), as well as the optimization of glycan linker length, and appreciate their inclusion in the manuscript. This manuscript clearly and concisely demonstrates a novel, generalizable method for the identification of proteolytic cleavages sites at the cell surface. The robust identification of a number of previously unidentified cleavages sites, as well as the identification of previously unidentified proteins at the cell surface, potentially due to previously unknown sheddase activity, is extremely promising.

Major comments:

1. It would be very helpful to specify that the glycans being used as handles here for stabiligase, are specifically sialic acid glycans. Not all terminal glycans bear the requisite diol that can be oxidized to become a reactive handle, a short description of the chemical reactivity of sialic acids here would also help to clarify the requirement for oxidation, and the effects observed using V. cholerae sialidase digestion.

2. Similarly, the CSC method (Wollscheid) also uses similar terminal sialic acid glycans as handles. When referring to glycosites, could the authors specific which (N- or O-linked) they are referring to? Terminal sialic acids can be present on both N-linked and O-linked glycoproteins. Glycolipids also bear terminal sialic acids.

3. In Fig. 2e, the +/- legend above the flow cytometry data is confusing, and inconsistent with previous legends. It is unclear which dataset belongs to which condition. We recommend a similar labeling strategy to Fig. 2c.

4. Could the authors discuss differences between general surfaceomes and new N-termini bit more: The observation from their previous work that there is broad remodeling of cell surfaces upon over-expression of KrasG12V, but that it somehow does not result in new N-termini is somewhat surprising (Fig. S8 flow cytometry data may not be appropriate to detect such changes – in fact, the histograms are subtly shifted). Could it be possible that over-expression of either KRAs or HER2 oncogenes also alter protease expression? Is KRas over-expression known to regulate HER2 and vice versa? What about changes in glycosylation?

5. Fig. 3e, while the caption notes that there is no significant differences in labeling efficiencies as a result of altering the glycan linker length, there does appear to be a steady decrease in overall biotin signal intensity as the linker length increases.

Minor comments:

1. In Fig. 3, the caption does not reflect the data presented in the figure. Fig. 3d (microscopy) is skipped, and Fig. 3e is described instead.

2. Are the Neo-N-termini identified necessarily due to proteolytic cleavage, or does this technique also label the N-termini of non-cleaved proteins? Presuming the technique labels both, this should be clarified, as “neo-N-termini” may suggest only cleaved proteins are identified.

3. While not necessary for publication, it would be interesting if the authors could detect the absence of specific proteases by a lack of different forms of proteolysis at the cell surface. Currently, we are observing cells at their “steady states,” with their neo-N-termini already generated instead of observing effects directly imparted by proteases. Although there is a short discussion of which proteases might be responsible for the detected N-termini, would it be possible to perform a direct experiment where a specific exogenous protease is added and the subsequent surfaceomes analyzed via this technique? Or perhaps addition of a specific protease inhibitor?

4. It would be helpful to note for the reader some limitations of the technique, including the dependence on sialic acid glycans. There are many cell states where sialic acid expression is lost.

Reviewer: 2

Comments to the Author

The manuscript by Schaefer and colleagues describes an interesting methodological advance for profiling the N-termini of proteins at the cell surface. Previously, the authors used a membrane-tethered version of their powerful subtiligase system to append tags to the N-termini of cell surface transmembrane proteins. However, a disadvantage of this system is that it requires heterologously introducing the subtiligase variant into cells. Here, the authors present an attractive alternative approach where they generate a subtiligase variant with an N-terminal nucleophile and then covalently attach this enzyme to the surface of cells via conjugation through a bridging bis-aminooxy/hydrazine reagent to extracellular glycans following treatment with an oxidant. The approach is shown to perform quite well and hundreds of N-termini are mapped, including those that differ across cell lines and between oncogenically transformed cells. The data are properly interpreted and offer initial insights into how specific oncogenes remodel cell surfaces to potentially support the malignant behavior of cancer

cells. The method should be of broad interest to chemical biologists, biochemists and cell biologists, and this Reviewer recommends acceptance of the manuscript after minor revision to address the points below.

Specific comments.

1. Is the N-terminal aminooxy/hydrazide-coupled subtiligase stable to storage or does it need to be made fresh for each experiment?

2. Line 109, Figure 2c callout should instead be Figure 2e.

3. Line 121, does “soluble subtiligase” refer to “unmodified subtiligase” in Figure 3b? In general, it would be helpful if the authors reviewed the text and figures to ensure that consistent terms are used in both settings, as this Reviewer struggled in certain places to relate the text to the results in the figures.

4. This Reviewer was also a bit confused by the term “neo-N-termini” and whether essentially all N-termini on the cell surface are considered “neo” due to signal peptide processing. The authors appear to oscillate between using the term neo-N-termini versus N-termini, and it might be helpful to readers to define at an early stage in the manuscript what neo-N-termini is intended to signify. The most unexpected results would appear to come from the large group of internal N-termini that are not resulting from predicted signal peptide cleavages, but this Reviewer can understand why the authors might prefer to refer to all cleaved N-termini (predicted or not) as “neo”.

5. Lines 260-261, in at least one instance where the authors compare their SILAC proteomic data to western blotting, it would be nice to show the SILAC quantification of different N-termini signals in Figure 5. This Reviewer had difficulty relating the heat map in panel d to the westerns in panel e, especially for proteins like NOTCH2 with multiple N-termini. Could the authors show a bar graph for the SILAC signals for these NOTCH2 N-termini in the three cell groups shown in Figure 5e, so that readers could compare that quantified mass spectrometry proteomics data to the Western signals?

6. The authors may want to acknowledge in the Conclusions that the requirement for oxidation of cell surfaces, while admittedly mild, may have the potential to confound some biological interpretations.

Author's Response to Peer Review Comments:

James A. Wells, Ph.D.
Professor in
Departments of
Pharmaceutical Chemistry
and Cellular & Molecular
Pharmacology

UCSF MC 2552
Byers Hall, Room 503A
1700 4th Street
San Francisco, CA
94158-2330

September 04, 2022

Dr. Editor
ACS Central Sciences

Dear Editor,

Thank you handling our manuscript "**Direct identification of proteolytic cleavages on living cells using a glycan tethered peptide ligase**" and inviting us to submit a revision that responds to the reviewers' comments. We were pleased that the reviewers judged our work to be significant, original, and of interest to the broad readership at *ACS Central Science*. We have addressed their concerns with a revised manuscript that is included with tracked changes.

A point-by-point response to each of the reviewers is provided below. We thank the reviewers for their careful reading of the manuscript and their helpful suggestions; we think the revision is stronger than the original as a result. I look forward to hearing from you.

Sincerely Yours,

James A. Wells, PhD
Harry and Dianna Hind Professor of Pharmaceutical Sciences

Point-by-point responses:

Reviewer(s)' Comments to Author:

Reviewer: 1

Recommendation: Publish in ACS Central Science after minor revisions noted.

Comments:
In this manuscript, the authors demonstrate the development of a novel tool for the labeling and subsequent identification of proteolytic cleavage sites at the cell surface. By tethering their engineered peptide ligase to extracellular glycans containing sialic acids, the authors distribute the enzyme across the cell surface, and subsequently label the N-termini of nearby proteins. After optimization, the authors demonstrate selective labeling of membrane proteins. Upon proteomic analysis, the authors identify several novel proteolytic cleavage sites across multiple biological contexts and proceed to validate the cleavage of several of these proteins across cancer cell lines with the HER2 and KRAS(G12V) mutations. The authors accomplished a well considered optimization of the N-terminomics labeling strategy. We especially note the consideration of multiple different strategies for labeling and tethering (multiple stabiligases, click-chemistry route), as well as the optimization of glycan linker length, and appreciate their inclusion in the manuscript. This manuscript clearly and concisely demonstrates a novel, generalizable method for the identification of proteolytic cleavages sites at the cell surface. The robust identification of a number of previously unidentified cleavages sites, as well as the identification of previously unidentified proteins at the cell surface, potentially due to previously unknown sheddase activity, is extremely promising.

We thank the reviewer for the thorough read of our manuscript and appreciate their recognition of the merit of our work.

Major comments:
1. It would be very helpful to specify that the glycans being used as handles here for stabiligase, are specifically sialic acid glycans. Not all terminal glycans bear the requisite diol that can be oxidized to become a reactive handle, a short description of the chemical reactivity of sialic acids here would also help to clarify the requirement for oxidation, and the effects observed using V. cholerae sialidase digestion.

We appreciate the reviewer's suggestions and also assume that the cis-diols on sialic acid are the most likely sites of oxidation under the mild sodium

periodate condition. To provide a more detailed chemical description, we added these sentences (lines 95-97):

"Glycan sugars containing diols, particularly sialic acid, are sensitive to mild periodate oxidation treatment and form aldehydes that can be coupled to aminooxy- and hydrazide- nucleophiles."

2. Similarly, the CSC method (Wollscheid) also uses similar terminal sialic acid glycans as handles. When referring to glycosites, could the authors specific which (N- or O-linked) they are referring to? Terminal sialic acids can be present on both N-linked and O-linked glycoproteins. Glycolipids also bear terminal sialic acids.

We determined the number of glycosites based on glycosylation annotations from Uniprot which included both N- and O- linked glycans. To provide clarity, we have edited the main text to include this description. We agree with the reviewer's comment that stabiligase can be attached to various sialic-acid containing glycans and avoid stating that stabiligase to attached to only glycoproteins.

3. In Fig. 2e, the +/- legend above the flow cytometry data is confusing, and inconsistent with previous legends. It is unclear which dataset belongs to which condition. We recommend a similar labeling strategy to Fig. 2c.

Per the reviewer's suggestion, we revised the legend of Fig. 2e to match the Fig. 2c legend.

4. Could the authors discuss differences between general surfaceomes and new N-termini bit more: The observation from their previous work that there is broad remodeling of cell surfaces upon over-expression of KrasG12V, but that it somehow does not result in new N-termini is somewhat surprising (Fig. S8 flow cytometry data may not be appropriate to detect such changes – in fact, the histograms are subtly shifted). Could it be possible that over-expression of either KRAs or HER2 oncogenes also alter protease expression? Is KRas over-expression known to regulate HER2 and vice versa? What about changes in glycosylation?

We appreciate the reviewer's comments that neo-N-termini differences in KRAS(G12V) and HER2 transformed cell lines are likely due to changes to the cell surface landscape including substrate abundance, protease expression, and the abundance of other PTMs. Our previous surfaceomics on KRAS(G12V) (Leung

et al, 2020) showed several up- and down- regulated proteases including ADAM proteases (ADAM -10 and -17 were downregulated, ADAM9 was upregulated), the downregulation of the serine protease matriptase, and upregulated endopeptidases (*e.g.*, ANPEP). Consistent with these observations, cell surface N-terminomics on KRAS(G12V) showed downregulated furin-cleavage at the pro-mature junctions of ADAM -10 and -17, and reduced matriptase shedding. We are keenly interested in defining protease-substrate relationships with cell surface N-terminomics, and are currently preparing experiments to remove or over-express cell surface proteases to profile neo-N-termini changes.

We cannot speak to a direct interaction between HER2 and KRAS(G12V) to our best knowledge, but as members of the MAPK pathway it is unsurprising that the two oncogenes result in some similarities to cell surface remodeling. In previous work (Leung et al, 2020), we also observed differences in glycosylation complexity and abundance across MCF10A oncogenic transformed cell lines and that glycosylation did not affect surfaceomics fold-changes. Along similar lines, we used flow cytometry in Fig. S8 to show that GT-stabiligase tethering was not impacted by glycosylation differences.

We have also included an additional discussion (lines 299-302) to address potential factors that may contribute to altered neo-N-termini:

"Proteases may be differently expressed under oncogenic transformations, but other factors such as protein interactions and additional PTMs may also influence cleavage events."

5. Fig. 3e, while the caption notes that there is no significant differences in labeling efficiencies as a result of altering the glycan linker length, there does appear to be a steady decrease in overall biotin signal intensity as the linker length increases.

Upon reanalysis, we agree that western blots with streptavidin show a very slight decrease in biotinylation with longer-linked glycan stabiligases. We have adjusted the description of Fig 3e.

Minor comments:

1. In Fig. 3, the caption does not reflect the data presented in the figure. Fig. 3d (microscopy) is skipped, and Fig. 3e is described instead.

We thank the reviewer for noticing this error and have added a caption for Figure 3d.

2. Are the Neo-N-termini identified necessarily due to proteolytic cleavage, or does this technique also label the N-termini of non-cleaved proteins?

Presuming the technique labels both, this should be clarified, as “neo-N-termini” may suggest only cleaved proteins are identified.

Yes, stabiligase exclusively labels accessible alpha-amines and except for initiator methionines, the ligase labels proteolysis sites.

3. While not necessary for publication, it would be interesting if the authors could detect the absence of specific proteases by a lack of different forms of proteolysis at the cell surface. Currently, we are observing cells at their “steady states,” with their neo-N-termini already generated instead of observing effects directly imparted by proteases. Although there is a short discussion of which proteases might be responsible for the detected N-termini, would it be possible to perform a direct experiment where a specific exogenous protease is added and the subsequent surfaceomes analyzed via this technique? Or perhaps addition of a specific protease inhibitor?

We agree with the reviewer’s excellent suggestions and we are very interested in pursuing these studies in future cell surface N-terminomics studies.

4. It would be helpful to note for the reader some limitations of the technique, including the dependence on sialic acid glycans. There are many cell states where sialic acid expression is lost.

We appreciate the reviewer’s comment that the diversity of sialic acid may impact stabiligase tethering. Surfaceomics (CSC), however, has been widely employed in 41 cell types (Bausch-Fluck et al, 2015) and we think GT-stabiligase tethering should be likewise compatible across these cells. Unlike CSC-based enrichment of glycoproteins, cell surface N-terminomics also requires much less glycan labeling (1 mM bioticin-hydrazide for CSC vs. 5 μM aminooxy-stabiligase). For cells lacking sialic acids, alternative glycan-oxidation methods such as the use of galactose oxidase to generate glycan-based aldehydes (Ramya et al, 2012) can enable stabiligase tethering. Based on the reviewer’s suggestion, we have included the additional discussion (lines 345-346):

“While GT-stabiligase was readily introduced in these cell types, cells such as those with sialic acid deficiency may require alternative oxidation strategies.”

Additional Questions:
Quality of experimental data, technical rigor: Top 5%

Significance to chemistry researchers in this and related fields: Top 5%

Broad interest to other researchers: Top 5%

Novelty: Top 5%

Is this research study suitable for media coverage or a First Reactions (a News & Views piece in the journal)?: Yes

Reviewer: 2

Recommendation: Publish in ACS Central Science after minor revisions noted.

Comments:
The manuscript by Schaefer and colleagues describes an interesting methodological advance for profiling the N-termini of proteins at the cell surface. Previously, the authors used a membrane-tethered version of their powerful subtiligase system to append tags to the N-termini of cell surface transmembrane proteins. However, a disadvantage of this system is that it requires heterologously introducing the subtiligase variant into cells. Here, the authors present an attractive alternative approach where they generate a subtiligase variant with an N-terminal nucleophile and then covalently attach this enzyme to the surface of cells via conjugation through a bridging bis-aminooxy/hydrazine reagent to extracellular glycans following treatment with an oxidant. The approach is shown to perform quite well and hundreds of N-termini are mapped, including those that differ across cell lines and between oncogenically transformed cells. The data are properly interpreted and offer initial insights into how specific oncogenes remodel cell surfaces to potentially support the malignant behavior of cancer cells. The method should be of broad interest to chemical biologists, biochemists and cell biologists, and this Reviewer recommends acceptance of the manuscript after minor revision to address the points below.

We appreciate the reviewer's comments and are glad that they enjoyed our work.

Specific comments.

1. Is the N-terminal aminooxy/hydrazide-coupled subtiligase stable to storage or does it need to be made fresh for each experiment?

N-terminal conjugated stabiligase aliquots were stored at -80˚C after purification. ESI-MS analysis confirmed stability after a freeze-thaw process. We avoided the use of glycerol during freezing because of the aminooxy- and hydrazide- groups. To avoid potential activity loss, aliquots were used only once following freeze-thaw. We have included this detail in the methods section regarding the preparation of conjugated stabiligases.

2. Line 109, Figure 2c callout should instead be Figure 2e.

We thank the reviewer for noticing this error and the correct Figure 2e is now called out.

3. Line 121, does "soluble subtiligase" refer to "unmodified subtiligase" in Figure 3b? In general, it would be helpful if the authors reviewed the text and figures to ensure that consistent terms are used in both settings, as this Reviewer struggled in certain places to relate the text to the results in the figures.

Yes, "soluble subtiligase" is also "unmodified subtiligase". We appreciate the reviewer's comment that interchangeable use of these terms is confusing, and revised the manuscript to exclusively describe unconjugated enzyme as "unmodified stabiligase".

4. This Reviewer was also a bit confused by the term "neo-N-termini" and whether essentially all N-termini on the cell surface are considered "neo" due to signal peptide processing. The authors appear to oscillate between using the term neo-N-termini versus N-termini, and it might be helpful to readers to define at an early stage in the manuscript what neo-N-termini is intended to signify. The most unexpected results would appear to come from the large group of internal N-termini that are not resulting from predicted signal peptide cleavages, but this Reviewer can understand why the authors might prefer to refer to all cleaved N-termini (predicted or not) as "neo".

We appreciate the reviewer's comments and have revised the manuscript to consistently refer to cell surface N-termini as neo-N-termini, since they are positioned after the initiator methionine.

5. Lines 260-261, in at least one instance where the authors compare their SILAC proteomic data to western blotting, it would be nice to show the SILAC quantification of different N-termini signals in Figure 5. This Reviewer had difficulty relating the heat map in panel d to the westerns in panel e, especially for proteins like NOTCH2 with multiple N-termini. Could the authors show a

bar graph for the SILAC signals for these NOTCH2 N-termini in the three cell groups shown in Figure 5e, so that readers could compare that quantified mass spectrometry proteomics data to the Western signals?

We appreciate the reviewer's comments regarding NOTCH2 proteolysis and have included a more detailed figure caption describing the cleavage bands observed in panel e. Unlike the other proteins in this panel, mature NOTCH2 is displayed on the cell surface as two non-covalently associated protein fragments, and is then further proteolyzed. During processing, the furin protease creates two fragments: one NOTCH2 fragment with a large extracellular domain (top band) that binds to another NOTCH2 fragment consisting of a small extracellular portion and a large intracellular region (middle band). The ADAM-protease cleaves NOTCH2 to form the third band on the western blot, a fragment containing a membrane-proximal extracellular nub with a large intracellular region. The neo-N-terminus generated by furin is upregulated and exclusively observed for KRAS(G12V). The ADAM-protease cleavage was represented by a neo-N-terminal peptide in both HER2 (upregulated) and KRASG12V (downregulated) datasets.

6. The authors may want to acknowledge in the Conclusions that the requirement for oxidation of cell surfaces, while admittedly mild, may have the potential to confound some biological interpretations.

We appreciate the reviewer's comment and although cells are rapidly oxidized on ice (500 μM sodium periodate, 10 min, 4 ˚C), we cannot rule out the possibility that small biological perturbations may arise. We note, however, that cell viability was not significantly affected. We acknowledge this possibility in the revised conclusion (lines 347-349).

Additional Questions:
Quality of experimental data, technical rigor: Top 5%

Significance to chemistry researchers in this and related fields: Top 5%

Broad interest to other researchers: Top 5%

Novelty: Top 5%

Is this research study suitable for media coverage or a First Reactions (a News & Views piece in the journal)?: No

oc-2022-00899w.R2

Name: Peer Review Information for "Direct identification of proteolytic cleavages on living cells using a glycan tethered peptide ligase"

Second Round of Reviewer Comments

Reviewer: 1

Comments to the Author

The authors have satisfactorily addressed concerns. This article will be a useful resource for those interested in surfaceome and proteolysis studies.

Reviewer: 2

Comments to the Author

The authors have done an excellent job addressing the Reviewer concerns. The manuscript is recommended with enthusiasm for acceptance.

Author's Response to Peer Review Comments:

September 08, 2022

Dr. Editor

ACS Central Science

Dear Editor,

Thank you for handling our manuscript so quickly. We are delighted that the manuscript is provisionally accepted in ACS Central Science. We are now submitting the formatting changes as requested to be officially accepted.

Sincerely Yours,

James A. Wells, PhD

Harry and Dianna Hind Professor of Pharmaceutical Sciences
